# Multiplatform molecular analyses refine classification of gliomas arising in patients with neurofibromatosis type 1

**DOI:** 10.1007/s00401-022-02478-5

**Published:** 2022-08-09

**Authors:** Calixto-Hope G. Lucas, Emily A. Sloan, Rohit Gupta, Jasper Wu, Drew Pratt, Harish N. Vasudevan, Ajay Ravindranathan, Jairo Barreto, Erik A. Williams, Anny Shai, Nicholas S. Whipple, Carol S. Bruggers, Ossama Maher, Burt Nabors, Michael Rodriguez, David Samuel, Melandee Brown, Jason Carmichael, Rufei Lu, Kanish Mirchia, Daniel V. Sullivan, Melike Pekmezci, Tarik Tihan, Andrew W. Bollen, Arie Perry, Anuradha Banerjee, Sabine Mueller, Nalin Gupta, Shawn L. Hervey-Jumper, Nancy Ann Oberheim Bush, Mariza Daras, Jennie W. Taylor, Nicholas A. Butowski, John de Groot, Jennifer L. Clarke, David R. Raleigh, Joseph F. Costello, Joanna J. Phillips, Alyssa T. Reddy, Susan M. Chang, Mitchel S. Berger, David A. Solomon

**Affiliations:** 1 Department of Pathology, University of California, San Francisco, 513 Parnassus Ave, Health Sciences West 451, San Francisco, CA 94143 USA; 2 Laboratory of Pathology, National Cancer Institute, National Institutes of Health, Bethesda, MD USA; 3 Department of Radiation Oncology, University of California San Francisco, San Francisco, CA USA; 4 Department of Neurological Surgery, University of California San Francisco, San Francisco, CA USA; 5 Division of Pediatric Hematology/Oncology, Department of Pediatrics, University of Utah, Salt Lake City, UT USA; 6 Department of Oncology, Nicklaus Children’s Hospital, Miami, FL USA; 7 Division of Neuro-Oncology, Department of Neurology, University of Alabama at Birmingham, Birmingham, AL USA; 8 Douglass Hanly Moir Pathology, Macquarie Park, Australia; 9 Department of Hematology/Oncology, Valley Children’s Hospital, Madera, CA USA; 10 Department of Neurosurgery, Valley Children’s Hospital, Madera, CA USA; 11 Department of Medical Genetics and Metabolism, Valley Children’s Hospital, Madera, CA USA; 12 Division of Pediatric Hematology/Oncology, Department of Pediatrics, University of California San Francisco, San Francisco, CA USA; 13 Department of Neurology, University of California San Francisco, San Francisco, CA USA; 14 Department of Pediatrics, University of California San Francisco, San Francisco, CA USA; 15 Division of Neuro-Oncology, Department of Neurological Surgery, University of California San Francisco, San Francisco, CA USA; 16 Present Address: Department of Pathology, Johns Hopkins University School of Medicine, Baltimore, MD USA; 17 Present Address: Department of Pathology, Medstar Georgetown University Hospital, Washington, DC USA

**Keywords:** Brain tumor, Astrocytoma, Glioma, Neurofibromatosis type 1, NF1, Selumetinib, Trametinib, Molecular neuropathology, Molecular neuro-oncology

## Abstract

Gliomas arising in the setting of neurofibromatosis type 1 (NF1) are heterogeneous, occurring from childhood through adulthood, can be histologically low-grade or high-grade, and follow an indolent or aggressive clinical course. Comprehensive profiling of genetic alterations beyond *NF1* inactivation and epigenetic classification of these tumors remain limited. Through next-generation sequencing, copy number analysis, and DNA methylation profiling of gliomas from 47 NF1 patients, we identified 2 molecular subgroups of NF1-associated gliomas. The first harbored biallelic *NF1* inactivation only, occurred primarily during childhood, followed a more indolent clinical course, and had a unique epigenetic signature for which we propose the terminology “pilocytic astrocytoma, arising in the setting of NF1”. The second subgroup harbored additional oncogenic alterations including *CDKN2A* homozygous deletion and *ATRX* mutation, occurred primarily during adulthood, followed a more aggressive clinical course, and was epigenetically diverse, with most tumors aligning with either high-grade astrocytoma with piloid features or various subclasses of IDH-wildtype glioblastoma. Several patients were treated with small molecule MEK inhibitors that resulted in stable disease or tumor regression when used as a single agent, but only in the context of those tumors with *NF1* inactivation lacking additional oncogenic alterations. Together, these findings highlight recurrently altered pathways in NF1-associated gliomas and help inform targeted therapeutic strategies for this patient population.

**Supplementary Information:**

The online version contains supplementary material available at 10.1007/s00401-022-02478-5.

## Introduction

Neurofibromatosis type 1 (NF1) is an autosomal dominant tumor predisposition syndrome characterized by the development of glial neoplasms of the central and peripheral nervous systems [15]. Primary gliomas of the CNS occur in approximately 20% of NF1 patients and are biologically heterogeneous [21, 31]. These CNS gliomas can occur in childhood through adulthood, arise throughout the neuroaxis including the optic pathway and spinal cord, may appear histologically low-grade or high-grade, and can follow an indolent or aggressive clinical course [13, 20, 22, 24]. Molecularly, these gliomas are driven by inactivation of the *NF1* tumor suppressor gene which encodes the neurofibromin protein that functions as a negative regulator of the RAS family of oncoproteins and downstream mitogen-activated protein (MAP) kinase signaling pathway [7, 16]. A recent genomics study of gliomas arising in the setting of NF1 identified additional oncogenic alterations in a subset, which included *ATRX* mutation, *CDKN2A* homozygous deletion, and *TP53* mutation [4]. However, comprehensive profiling of additional genetic alterations beyond *NF1* has not been widely performed and definitive epigenetic classification of gliomas arising in the setting of NF1 has not been reported. Whether these NF1-associated gliomas represent distinct biologic entities and follow different clinical courses with unique therapeutic vulnerabilities compared to sporadic gliomas with shared histologic diagnosis is poorly understood.

Recent advances in diagnostic and bioinformatics technology have allowed for epigenetic classification of brain tumors using unsupervised hierarchical clustering analysis and other machine learning methods [3]. This analysis allows for more accurate integrated diagnosis than routine histopathologic assessment alone. Given the wide histologic spectrum of NF1-associated gliomas, improved stratification schemes incorporating histologic, genetic, and epigenetic features will better guide management for this patient population. To address this need, we have assembled a cohort of 47 gliomas arising in the setting of NF1 and used multiplatform molecular profiling to develop a new classification scheme with prognostic and therapeutic significance.

## Methods

### Patient cohort

The study cohort consisted of 47 patients with neurofibromatosis type 1 (NF1) who underwent surgical sampling of a glioma (Supplementary Table 1 [Online Resource 1]). The diagnosis of NF1 was established either by: 1) sequencing of a normal constitutional DNA sample isolated from peripheral blood or buccal swab demonstrating a deleterious/pathogenic mutation or deletion of the *NF1* gene, or 2) the combination of established clinical diagnostic criteria [19] along with tumor-only genomic profiling revealing biallelic *NF1* inactivation (*i.e.,* single *NF1* mutation with allele frequency > 50% consistent with a heterozygous germline event combined with loss of heterozygosity, or alternatively two or more *NF1* mutations with one present at ≥ 50% allele frequency consistent with representing a heterozygous germline event). This study complied with all relevant ethical regulations and was approved by the UCSF Institutional Review Board.

### Histopathologic review of glioma specimens

Detailed pathologic examination was retrospectively performed on the entire glioma cohort to investigate their histologic and immunohistochemical features. Representative hematoxylin and eosin (H&E) stained sections and immunohistochemical stains from the 47 gliomas were digitally scanned on an Aperio slide scanner to assemble a digital pathology library composed of 345.SVS image files (308 Gigabytes of histology data) from which histologic and immunohistochemical features were reviewed and annotated using ImageScope software (Leica Biosystems). Histologic features that were individually annotated for each glioma include predominant growth pattern, cellular density, cellular morphology, cytoplasmic features, stroma, multinucleated cells, Rosenthal fibers, eosinophilic granular bodies, calcifications, perivascular inflammatory infiltrate, hemosiderin deposits, dysmorphic ganglion cell component, mitoses per 2 mm^2^, vascular endothelial proliferation, and necrosis. An overall histologic impression for each glioma was made as either pilocytic astrocytoma, ganglioglioma, diffuse astrocytoma, anaplastic pilocytic astrocytoma, or high-grade astrocytoma. The histologic designation of anaplastic pilocytic astrocytoma was used for those gliomas with piloid features including Rosenthal fibers that had elevated mitotic activity (> 3 mitoses per 2 mm^2^) with or without necrosis. The histologic designation of high-grade astrocytoma was used for those gliomas lacking Rosenthal fibers that had elevated mitotic activity (> 3 mitoses per 2 mm^2^) with or without necrosis.

Immunohistochemistry was performed on whole formalin-fixed, paraffin-embedded tissue sections using the following antibodies: glial fibrillary acidic protein (GFAP, DAKO, polyclonal, 1:3,000 dilution, no antigen retrieval), OLIG2 (Immuno Bio Labs, polyclonal, 1:200 dilution, ER1 antigen retrieval), SOX10 (Cell Marque, clone EP268, dilution 1:250, ER2 antigen retrieval), synaptophysin (Cell Marque, polyclonal, 1:100 dilution, ER2 antigen retrieval), p16 (MTM Labs, clone E6H4, undiluted, ER1 antigen retrieval), ATRX (Sigma, polyclonal, 1:100 dilution), p53 (Biocare, clone DO-7, 1:100 dilution, ER2 antigen retrieval), histone H3 K27M mutant protein (RevMAb Biosciences, clone RM192, 1:600 dilution), histone H3 lysine 27 trimethylation (Cell Signaling, cat #9733, clone C36B11, 1:50 dilution, ER2 antigen retrieval), and Ki-67 (Dako, clone Mib1, 1:50 dilution, ER2 antigen retrieval). Immunostaining was performed in Leica Bond-Max or Ventana BenchMark Ultra automated stainers.

### Targeted next-generation DNA sequencing analysis

Tumor tissue was selectively scraped from unstained slides or punched from formalin-fixed, paraffin-embedded blocks using biopsy punches (Integra Miltex Instruments) to enrich for maximal tumor content. Genomic DNA was extracted from this macro-dissected formalin-fixed, paraffin-embedded tumor tissue using the QIAamp DNA FFPE Tissue Kit (Qiagen). A normal constitutional DNA sample was also extracted from a peripheral blood or buccal swab specimen for 21 of the patients using the QIAamp DNA Blood Midi Kit (Qiagen). Targeted next-generation sequencing was performed using the UCSF500 NGS Panel as previously described [18]. Capture-based next-generation DNA sequencing was performed using an assay that targets all coding exons of 479 cancer-related genes, select introns and upstream regulatory regions of 47 genes to enable detection of structural variants including gene fusions, and DNA segments at regular intervals along each chromosome to enable genome-wide copy number and zygosity analysis, with a total sequencing footprint of 2.8 Mb (Supplementary Table 2 [Online Resource 1]). Multiplex library preparation was performed using the KAPA Hyper Prep Kit (Roche) according to the manufacturer’s specifications. Hybrid capture of pooled libraries was performed using a custom oligonucleotide library (Nimblegen SeqCap EZ Choice). Captured libraries were sequenced as paired-end reads on an Illumina NovaSeq 6000 instrument. Sequence reads were mapped to the reference human genome build GRCh37 (hg19) using the Burrows-Wheeler aligner (BWA). Recalibration and deduplication of reads was performed using the Genome Analysis Toolkit (GATK). Coverage and sequencing statistics were determined using Picard CalculateHsMetrics and Picard CollectInsertSizeMetrics. Single nucleotide variant and small insertion/deletion mutation calling was performed with FreeBayes, Unified Genotyper, and Pindel. Large insertion/deletion and structural alteration calling was performed with Delly. Variant annotation was performed with Annovar. Single nucleotide variants, insertions/deletions, and structural variants were visualized and verified using Integrative Genome Viewer. Genome-wide copy number and zygosity analysis was performed by CNVkit and visualized using NxClinical (Biodiscovery).

### DNA methylation profiling analysis

Genomic DNA was bisulfite converted using the EZ DNA Methylation kit following the manufacturer’s recommended protocol (Zymo Research). Bisulfite converted DNA was then amplified, fragmented, and hybridized to Infinium EPIC 850k Human DNA Methylation BeadChips following the manufacturer’s recommended protocol (Illumina). Methylation data were preprocessed using the combineArrays function within the minfi package (v.1.38.0) in R Bioconductor (version 3.5.3) [1]. The detection *p* value for each sample was computed, and CpG sites with detection *p* values above 0.05 were discarded from the analysis. Functional normalization with NOOB background correction and dye-bias normalization was performed [9, 29]. Probe filtering was performed after normalization. Specifically, probes located on sex chromosomes, containing nucleotide polymorphisms (dbSNP132 Common) within five base pairs of and including the targeted CpG site, or mapping to multiple sites on hg19 (allowing for one mismatch), as well as cross reactive probes were removed from analysis.

The DNA methylation profiles of the NF1-associated tumors were assessed together with 1561 reference samples spanning 39 CNS tumor methylation groups and 7 control tissue methylation groups previously generated at DKFZ or UCSF (sample manifest in Supplementary Table 3 [Online Resource 1]) [3, 14, 26]. These included 78 A IDH (astrocytoma, IDH-mutant), 46 A IDH-HG (astrocytoma, IDH-mutant, high-grade), 21 ANA PA (anaplastic astrocytoma with piloid features), 12 CHGL (chordoid glioma), 21 CN (central neurocytoma), 8 CONTR CEBM (control cerebellum), 13 CONTR HEMI (control hemisphere), 9 CONTR HYPTHAL (control hypothalamus), 24 CONT INFLAM (control inflammation), 12 CONTR PONS (control pons), 23 CONTR REACT (control reactive), 9 CONTR WM (control white matter), 8 DLGNT (diffuse leptomeningeal glioneuronal tumor), 78 DMG-K27 (diffuse midline glioma, H3 K27M-mutant), 28 EPN MPE (ependymoma, myxopapillary), 91 EPN PFA (ependymoma, posterior fossa type A), 51 EPN PFB (ependymoma, posterior fossa type B), 70 EPN RELA (ependymoma, RELA-fused), 27 EPN SPINE (ependymoma, spinal), 11 EPN YAP (ependymoma, YAP-fused), 22 EVN (extraventricular neurocytoma), 41 GBM G34 (diffuse hemispheric glioma, H3 G34-mutant), 56 GBM MES (glioblastoma, IDH-wildtype, mesenchymal subclass), 14 GBM MID (glioblastoma, IDH-wildtype, midline subclass), 16 GBM MYCN (glioblastoma, IDH-wildtype, MYCN subclass), 64 GBM RTK1 (glioblastoma, IDH-wildtype, RTK1 subclass), 143 GBM RTK2 (glioblastoma, IDH-wildtype, RTK2 subclass), 13 GBM RTK3 (glioblastoma, IDH-wildtype, RTK3 subclass), 23 HGNET BCOR (high-grade neuroepithelial tumor, BCOR-altered), 21 HGNET MN1 (high-grade neuroepithelial tumor, MN1-altered), 10 IHG (infant type hemispheric glioma), 8 DIG/DIA (desmoplastic infantile ganglioglioma/astrocytoma), 44 DNT (dysembryoplastic neuroepithelial tumor), 21 GG (ganglioglioma), 22 LGG MYB (low-grade glioma, MYB/MYBL1 fusion positive), 38 PA MID (pilocytic astrocytoma, midline subclass), 114 PA PF (pilocytic astrocytoma, posterior fossa subclass), 24 PA ST (pilocytic astrocytoma, supratentorial subclass), 9 RGNT (rosette-forming glioneuronal tumor), 21 SEGA (subependymal giant cell astrocytoma), 80 O IDH (oligodendroglioma, IDH-mutant and 1p/19q-codeleted), 8 PLNTY (polymorphous low-grade neuroepithelial tumor of the young), 44 PXA (pleomorphic xanthoastrocytoma), 37 SUBEPN PF (subependymoma, poster fossa), 9 SUBEPN SPIN (subependymoma, spinal), and 19 SUBEPN ST (subependymoma, supratentorial). Since a subset of the reference cohort contained methylation data generated using the Infinium Human Methylation 450k BeadChips, the approximately 450,000 overlapping CpG sites between the EPIC 850k and 450k BeadChips were used in the analysis. A beta value matrix with approximately 384,000 CpG probes was used for all downstream analysis. Row-wise standard deviation was calculated for each probe across all samples, and the 35,000 most differentially methylated probes were selected. Dimensionality reduction using t-distributed stochastic neighbor embedding (tSNE) was performed by Rtsne (v.0.15) using the following analysis parameters: dims = 2, max_iter = 5000, theta = 0, perplexity = 28. The tSNE plot was visualized with ggplot2 (v.3.3.5) [http://ggplot2.tidyverse.org/].

Unsupervised hierarchical clustering was performed with the hclust function in Rstats (v.3.6.0) to assess variation in DNA methylation patterns and determine any relevant epigenetic subgrouping among the NF1-associated gliomas from this study alongside the ANA PA, PA PF, PA MID, and PA ST reference clusters previously generated at DKFZ (sample manifest in Supplementary Table 4 [Online Resource 1]) [3]. The lmFit function from the Limma package (v.3.40.6) was applied on a log-transformed *b* value matrix to identify the 2,000 most differentially methylated CpG probes across the tumor cohort. Then K-means clustering utilizing the Pearson distance matrix with complete linkage was used to determine the optimal number of clusters, through 500 re-sampling interactions of the dataset for K-means of 2 through 10. Visualization was performed using the R package ComplexHeatmap (v.2.8.0) [12].

### MEK inhibitor treatment

An FDA-approved small molecule MEK inhibitor (either trametinib or selumetinib) was used off-label to treat nine patients in this study. Recommended age-appropriate dosing was followed, and interval assessment for known toxicities (*e.g.,* skin rash) and treatment response was performed.

### Statistical analysis

Statistical comparison of clinicopathologic features was performed by Mann–Whitney unpaired two-tailed t test using GraphPad Prism. Clinical outcomes were studied by Kaplan–Meier analysis using GraphPad Prism and R version 4.0 (http://www.r-project.org/). The Kaplan–Meier survival analysis was stratified by molecular group, epigenetic subgroup, and histologic features. For stratification of clinical outcomes by histologic features, gliomas were grouped as histologically low-grade (overall morphologic impression of pilocytic astrocytoma, ganglioglioma, or diffuse astrocytoma) or histologically high-grade (overall morphologic impression of anaplastic pilocytic astrocytoma or high-grade astrocytoma). Survival analysis *p* values were calculated by Log-rank (Mantel-Cox) test. Glioma-specific survival was defined as the time from initial diagnostic surgical procedure for the glioma until death due specifically to glioma progression or last clinical follow-up visit. Patients who died of causes unrelated to the glioma (*e.g.,* malignant peripheral nerve sheath tumor) were censored from the glioma-specific survival analysis at time of death.

## Results

### Gliomas arising in the setting of neurofibromatosis type 1 are driven by biallelic NF1 inactivation via diverse mechanisms

**Fig. 1:**
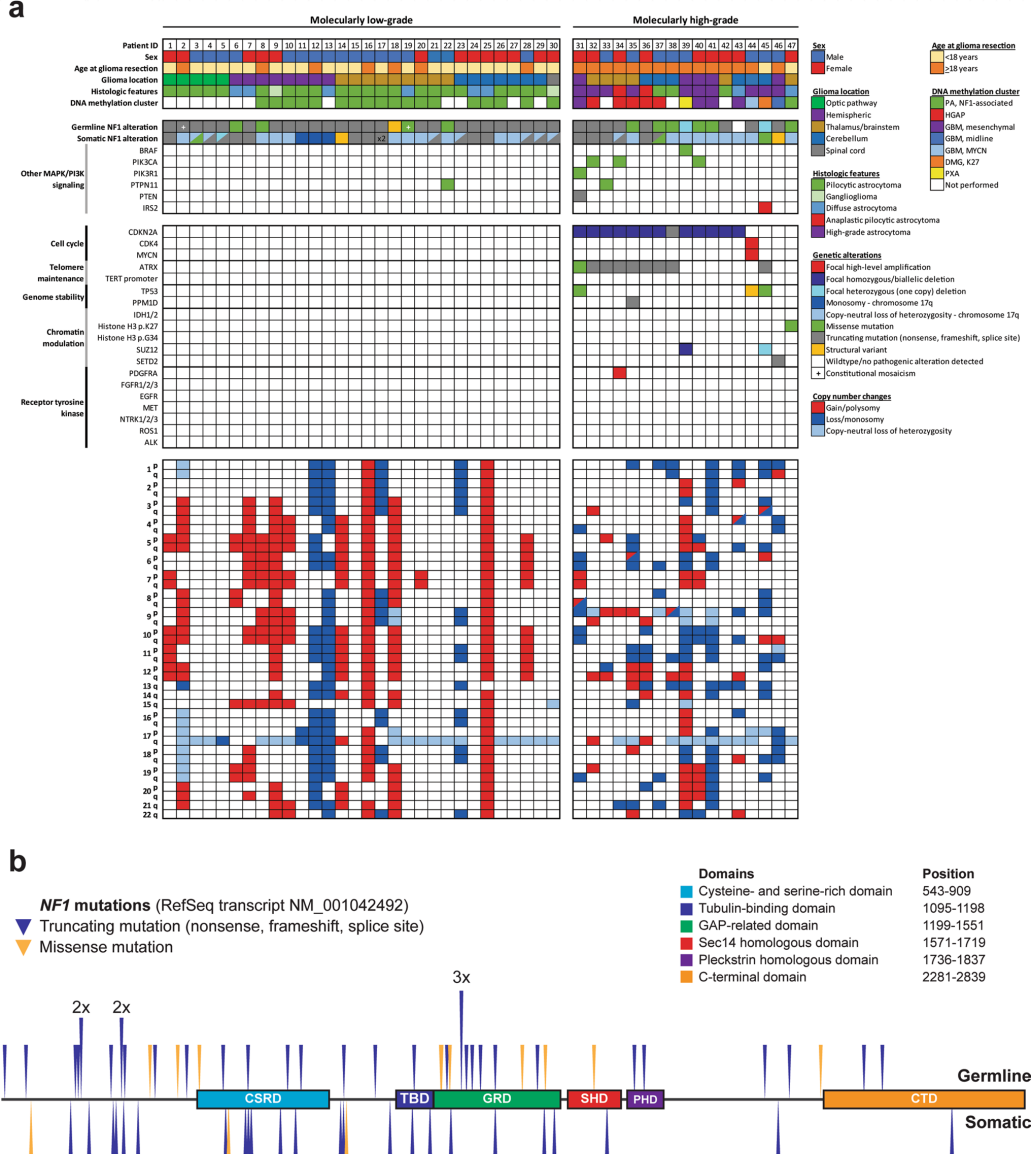
**a** Oncoprint table summarizing the clinical characteristics, histologic features, DNA methylation cluster assignments, genetic alterations, and chromosomal copy number aberrations identified in the 47 gliomas arising in patients with neurofibromatosis type 1. **b** Germline and somatic *NF1* gene mutations identified in the 47 gliomas arising in patients with neurofibromatosis type 1

Next-generation sequencing of gliomas arising in 47 NF1 patients revealed biallelic inactivation of the *NF1* gene in all tumors (Fig. 1a, Supplementary Tables 5–7 [Online Resource 1]). The majority of *NF1* mutations were truncating events (*e.g.,* nonsense, frameshift, or splice site), with a lesser number of missense substitutions that were scattered across the coding sequence (Fig. 1b). In the 21 patients with sequencing that was performed on a dedicated normal constitutional DNA sample, 19 demonstrated a pathogenic/deleterious mutation or deletion of the *NF1* gene on chromosome 17q11.2 that was present at approximately 50% allele frequency consistent with a heterozygous germline event present on one of two alleles. In the other two patients (#2 and #19), the normal DNA sample contained a pathogenic/deleterious mutation of the *NF1* gene at 37% and 38% allele frequencies, indicative of constitutional mosaicism due to a post-zygotic mutational event that occurred in utero.

Consistent with an autosomal dominant tumor predisposition syndrome, these gliomas arising in the setting of NF1 developed in patients with a heterozygous germline mutation or deletion involving one of two *NF1* alleles (apart from patients #2 and #19), with tumors that developed following somatic inactivation of the remaining wildtype allele through either loss of heterozygosity (LOH) or a second tumor-acquired mutation. In 36 of the 47 gliomas, the second *NF1* allele was somatically inactivated by a single event, most often via LOH (*n* = 22) or less frequently via truncating or missense mutation (*n* = 12) or structural rearrangement (*n* = 2). The other 11 gliomas demonstrated two or more somatic events contributing to inactivation of the remaining wild-type *NF1* allele. For example, patient #34 had a germline *NF1* p.R1276* nonsense mutation which was present at approximately 50% allele frequency in the normal constitutional DNA sample. The glioma in this patient had somatic inactivation of the remaining wild-type allele via two different mechanisms: c.4110 + 2 T > G splice site mutation present at 24% allele frequency and also LOH of chromosome 17q resulting in the germline p.R1276* nonsense mutation being present at 60% allele frequency.

### Genomic profiling reveals two clinically divergent molecular subgroups of NF1-associated gliomas

Two distinct molecular groups were identified among the 47 NF1-associated gliomas (Fig. 1a). The first group (“molecular low-grade”) consisted of those 29 tumors with biallelic inactivation of the *NF1* tumor suppressor gene only and one tumor with biallelic inactivation of *NF1* plus an activating missense mutation in *PTPN11*, a phosphatase that regulates the MAP kinase signaling pathway. This molecular low-grade group of NF1-associated gliomas was genetically defined by activation of the MAP kinase signaling pathway in isolation without additional oncogenic alterations affecting cell cycle regulatory factors (*e.g., CDKN2A*, *CDK4*), telomere maintenance genes (*e.g., TERT*, *ATRX*), chromatin modulation (*e.g., IDH1/2*, histone H3 isoforms, *SETD2*), or receptor tyrosine kinases (*e.g., EGFR*, *PDGFRA*, and *FGFR1*). The second group (“molecular high-grade”) consisted of those 17 tumors with biallelic inactivation of the *NF1* tumor suppressor gene plus additional oncogenic alterations that most commonly included *CDKN2A* mutation or homozygous deletion (13/17, 76%), *ATRX* mutation (9/17, 53%), *PIK3CA* or *PIK3R1* mutation (4/17, 24%), *TP53* mutation (3/17, 18%), and *SUZ12* deletion (2/17, 12%). None of the 47 NF1-associated gliomas in this cohort had *IDH1* or *IDH2* mutation, *EGFR* amplification, *TERT* promoter mutation, or histone H3 p.G34 mutation. Assessment of chromosomal copy number aberrations revealed frequent gains or losses of whole chromosomes in gliomas belonging to the molecular low-grade group, whereas gliomas in the molecular high-grade group often had gains/losses involving chromosome arms or interstitial segments of chromosomes. There was also monosomy or copy-neutral loss of heterozygosity of chromosome 17q in the majority of gliomas across both molecular groups that functioned as the mechanism for eliminating the remaining wild-type *NF1* allele located at 17q11.2.

**Fig. 2:**
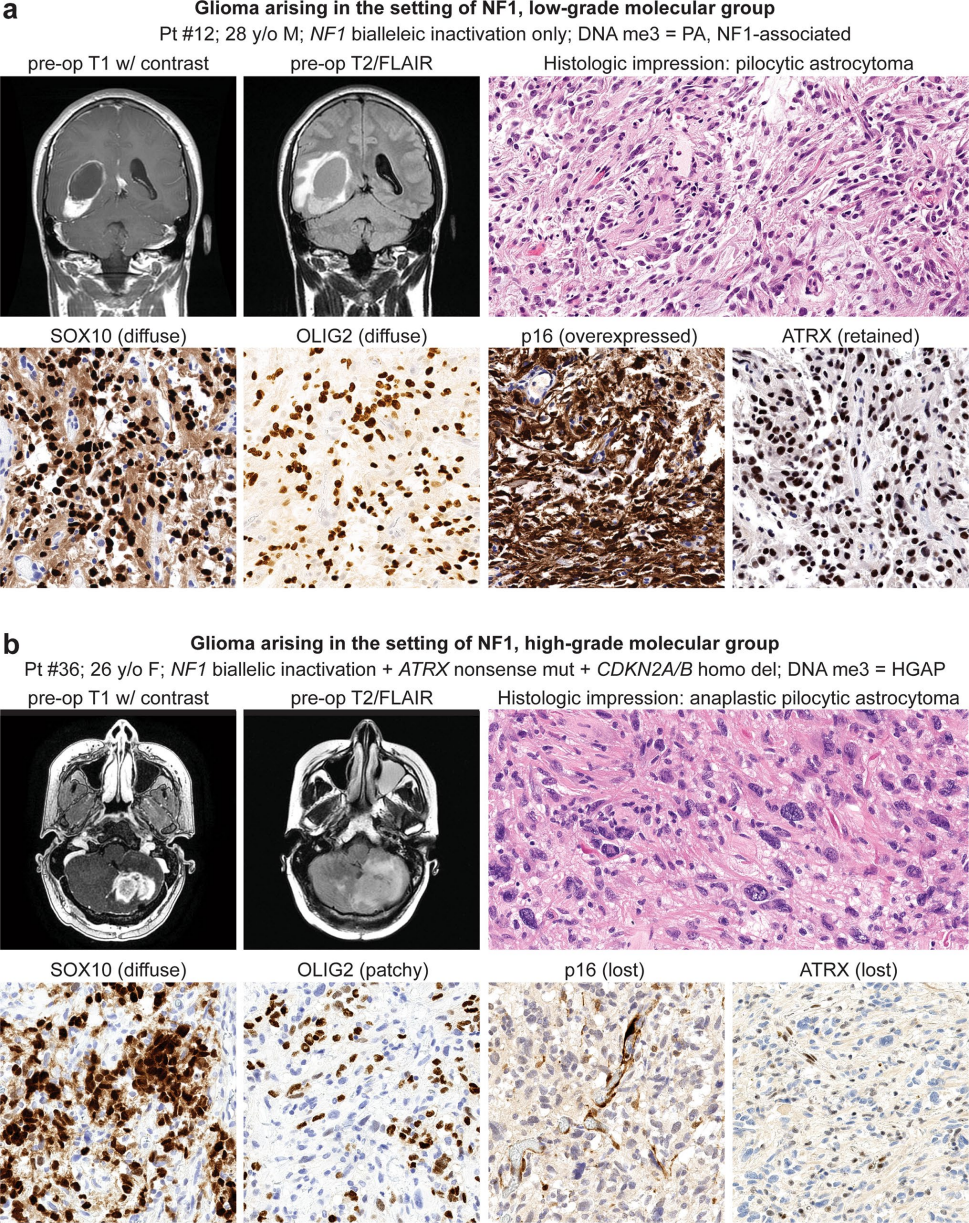

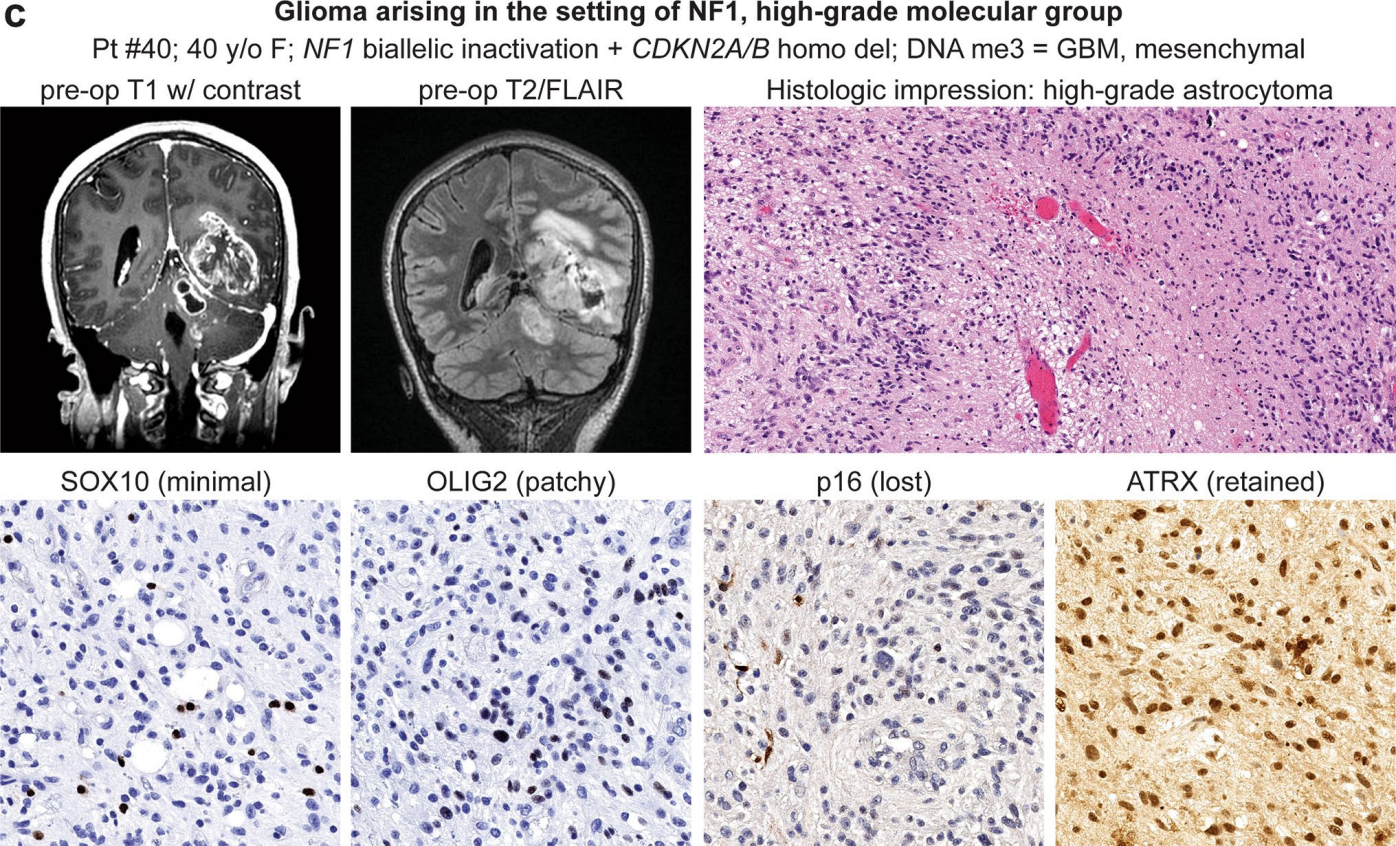
Imaging and histologic features of gliomas arising in patients with neurofibromatosis type 1. **a** Pilocytic astrocytoma, NF1-associated. **b** High-grade astrocytoma with piloid features, NF1-associated. **c** Glioblastoma, NF1-associated

The molecular low-grade group consisted of 30 patients (17 males, 13 females) with a median age of 12.4 years (Table 1, Fig. 2a). These tumors were located throughout the neuroaxis including optic pathway (17%), cerebral hemispheres (27%), thalamus/brainstem (30%), cerebellum (23%), and spinal cord (3%) (Supplementary Fig. 1 [Online Resource 2]). These tumors most often had histologic features resembling pilocytic astrocytoma (73%), but a subset also resembled diffuse astrocytoma (20%) or ganglioglioma (7%) (Supplementary Tables 8–9 [Online Resource 1], Supplementary Fig. 2 [Online Resource 2]). In contrast, the molecular high-grade group consisted of 17 patients (8 males, 9 females) with a median age of 28.3 years. These tumors were located in the cerebral hemispheres (30%), thalamus/brainstem (35%), and cerebellum (35%), with none occurring in the optic pathway or spinal cord. The majority of these tumors histologically resembled either anaplastic pilocytic astrocytoma (12%) or high-grade astrocytoma (58%) (Fig. 2b, c), but a subset also had low-grade histologic features lacking elevated mitotic activity or necrosis and resembling either pilocytic astrocytoma (18%) or diffuse astrocytoma (12%). NF1-associated gliomas in the molecular high-grade group often had increased cellular density, higher Ki-67 labeling indices, and loss of p16 and/or ATRX expression on immunohistochemical evaluation compared to the molecular low-grade group.

**Table 1:** Characteristics of 47 patients with gliomas arising in the setting of neurofibromatosis type 1 stratified by molecular low-grade versus high-grade groups

	Total cohort	Tumor molecular group		*p* value
		Low-grade	High-grade	
Patient sex				
Male	53% (25/47)	57% (17/30)	47% (8/17)	0.56
Female	47% (22/47)	43% (13/30)	53% (9/17)	0.56
Age at glioma surgery				
Median (years)	15.8	12.4	28.3	**0.0001**
Interquartile range (years)	11–27.7	8.2–6.2	20.3–35.7	
Glioma location				
Optic pathway	10% (5/47)	17% (5/30)	0% (0/17)	0.14
Cerebral hemispheres	28% (13/47)	27% (8/30)	30% (5/17)	0.99
Thalamus/brainstem	32% (15/47)	30% (9/30)	35% (6/17)	0.75
Cerebellum	28% (13/47)	23% (7/30)	35% (6/17)	0.50
Spinal cord	2% (1/47)	3% (1/30)	0% (0/17)	0.99
Histologic features				
Pilocytic astrocytoma	53% (25/47)	73% (22/30)	18% (3/17)	**0.0003**
Ganglioglioma	4% (2/47)	7% (2/30)	0% (0/17)	0.53
Diffuse astrocytoma	17% (8/47)	20% (6/30)	12% (2/17)	0.69
Anaplastic pilocytic astrocytoma	4% (2/47)	0% (0/30)	12% (2/17)	0.13
High-grade astrocytoma	22% (10/47)	0% (0/30)	58% (10/17)	** < 0.0001**
Genetic alterations				
* NF1* mutation/deletion	100% (47/47)	100% (30/30)	100% (17/17)	0.99
* CDKN2A* mutation/deletion	28% (13/47)	0% (0/30)	76% (13/17)	** < 0.0001**
* ATRX* mutation	19% (9/47)	0% (0/30)	53% (9/17)	** < 0.0001**
* TP53* mutation	6% (3/47)	0% (0/30)	18% (3/17)	**0.041**
* IDH1/2* mutation	0% (0/47)	0% (0/30)	0% (0/17)	0.99
Histone H3 mutation	2% (1/47)	0% (0/30)	6% (1/17)	0.36
* TERT* promoter mutation	0% (0/47)	0% (0/30)	0% (0/17)	0.99
* EGFR* amplification	0% (0/47)	0% (0/30)	0% (0/17)	0.99
* PTEN* mutation/deletion	2% (1/47)	0% (0/30)	6% (1/17)	0.36
DNA methylation cluster				
PA, NF1-associated	59% (19/32)	100% (18/18)	7% (1/14)	** < 0.0001**
HGAP	16% (5/32)	0% (0/18)	36% (5/14)	**0.009**
GBM, IDH-wildtype	19% (6/32)	0% (0/18)	43% (6/14)	**0.003**
PXA	3% (1/32)	0% (0/18)	7% (1/14)	0.44
DMG, H3 K27	3% (1/32)	0% (0/18)	7% (1/14)	0.44
Glioma extent of resection				
Biopsy	34%(16/47)	40% (12/30)	23% (4/17)	0.34
Subtotal	34%(16/47)	20% (6/30)	59% (10/17)	**0.011**
Gross total	32%(15/47)	40% (12/30)	18% (3/17)	0.19
Adjuvant radiation therapy				
%	35% (15/43)	14% (4/29)	79% (11/14)	** < 0.0001**
Adjuvant chemotherapy				
%	42% (18/43)	28% (8/29)	71% (10/14)	**0.009**
Glioma progression				
%	38% (18/47)	23% (7/30)	65% (11/17)	**0.011**
Median (mos)	16.1	23.5	7.4	
Glioma-related death				
%	17% (8/47)	0% (0/30)	47% (8/17)	** < 0.0001**
Median (mos)	12.7	n/a	12.7	

Bolded *p* values indicate significant differences between the low-grade vs. high-grade molecular groups

**Fig. 3:**
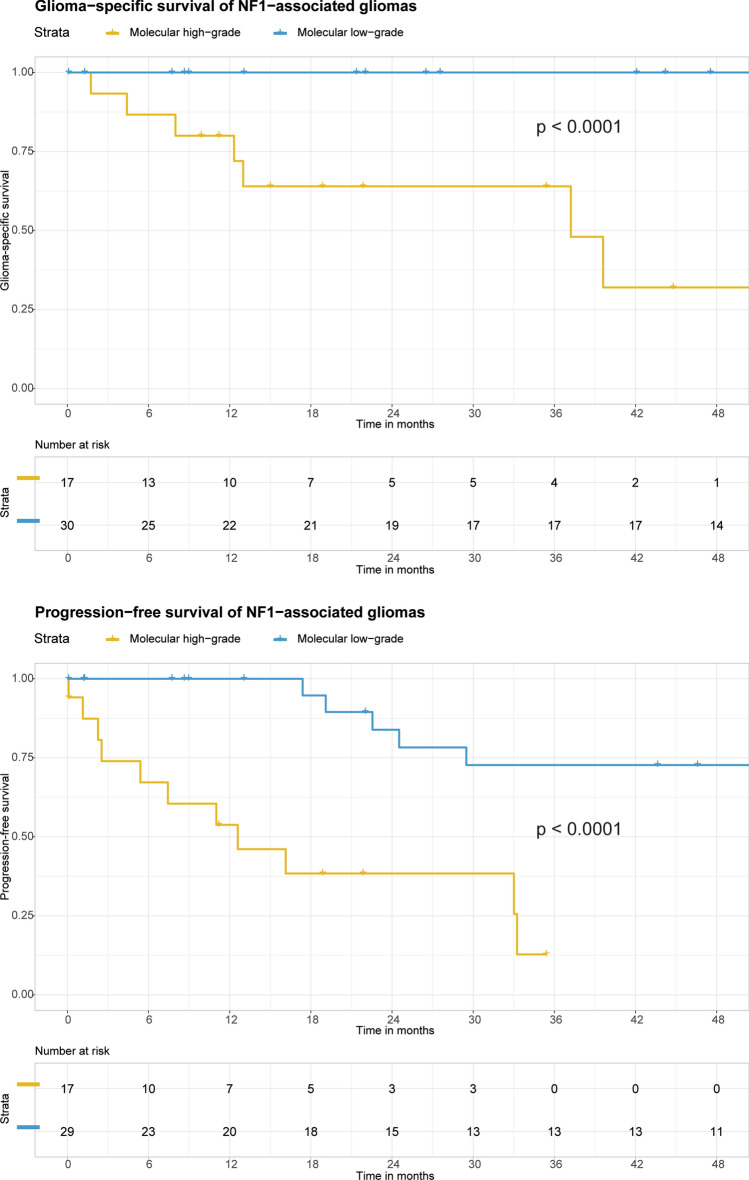
Kaplan–Meier curves comparing glioma-specific survival (**a**) and progression-free survival (**b**) for the 47 NF1 patients stratified by molecular low-grade versus high-grade groups

Clinical data for the patient cohort including manifestations of neurofibromatosis type 1, treatment, and outcomes are provided in Supplementary Table 1 [Online Resource 1]. Kaplan–Meier analysis of patient outcomes stratified by the two molecular groups revealed significantly inferior outcomes for the molecular high-grade group for both glioma-specific survival and progression-free survival (Fig. 3). At time of last clinical follow-up, no patients belonging to the molecular low-grade group had died due to glioma progression. In comparison, Kaplan–Meier analysis of patient outcomes stratified by microscopic features as either histologically low-grade or high-grade also revealed significantly inferior glioma-specific survival and progression-free survival for the histologically high-grade group (Supplementary Fig. 3 [Online Resource 2]). However, multiple patients with histologically low-grade gliomas died due to glioma progression during the period of clinical follow-up, specifically three of the five patients with histologically low-grade gliomas that belonged to the molecular high-grade group.

### Treatment with MEK inhibitors for patients with NF1-associated gliomas

**Fig. 4:**
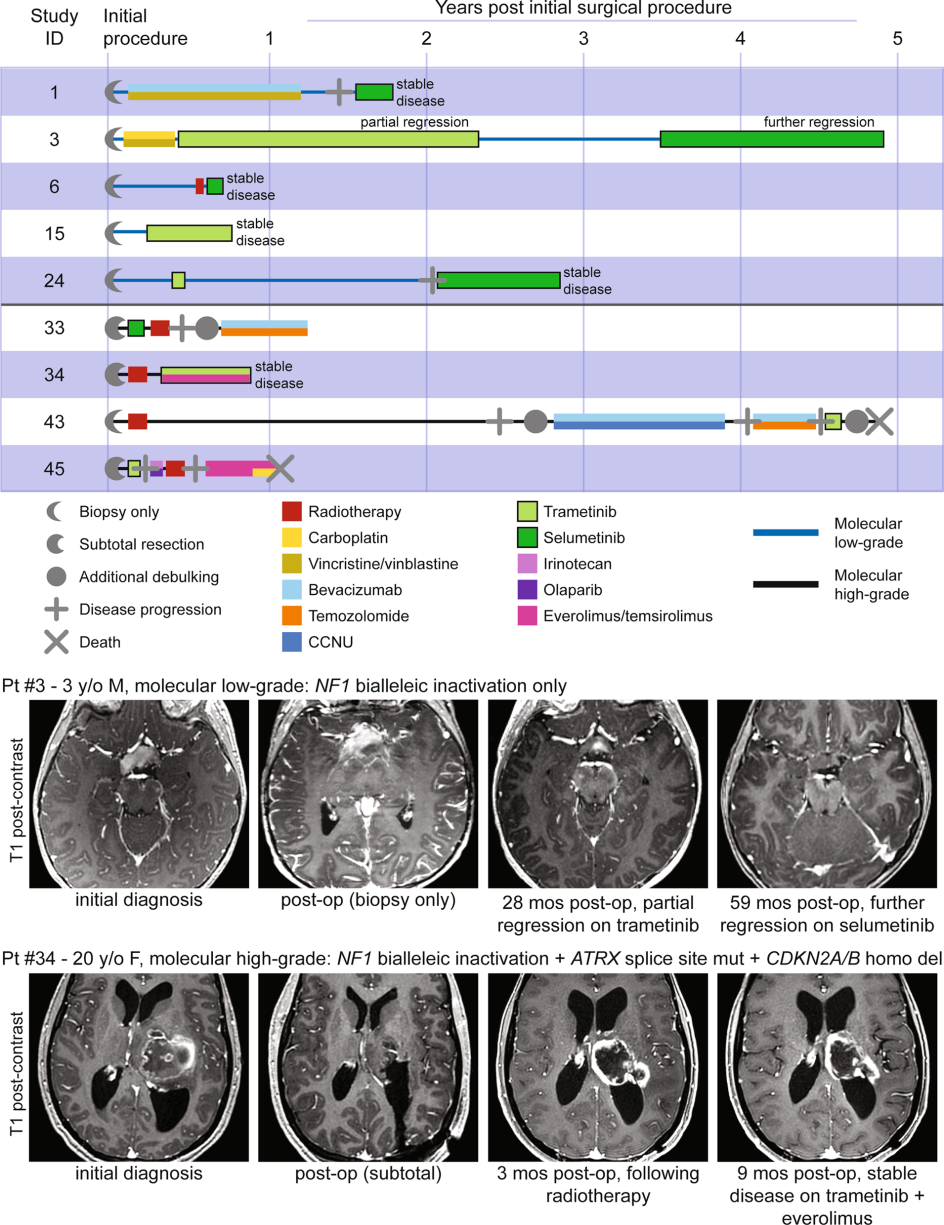
Clinical outcomes for patients with NF1-associated gliomas treated with MEK inhibitors. Swimmer’s plot (top) showing treatment course and clinical outcomes for nine NF1-associated glioma patients whose treatment included either trametinib or selumetinib. Imaging (bottom) demonstrating tumor regression for patient #3 and stable disease for patient #34 while on MEK inhibitor therapy

Given the primary role of *NF1* as a negative regulator of Ras/MAPK signaling, nine patients in this cohort were treated with a small molecule MEK inhibitor, either trametinib or selumetinib, during their disease course (Fig. 4). Of the five patients with NF1-associated gliomas belonging to the molecular low-grade group, four experienced stable disease and absence of tumor growth while on MEK inhibitor therapy at time of last clinical follow-up. The fifth patient (#3) experienced partial regression of an optic pathway glioma during an initial treatment course with trametinib and then further regression during a subsequent treatment course with selumetinib. Of the four patients with NF1-associated gliomas belonging to the molecular high-grade group, three demonstrated glioma progression during treatment with MEK inhibitor as a single agent. The fourth patient experienced stable disease and absence of tumor growth while on the combination of trametinib and everolimus (a small molecule mTOR inhibitor). No patients experienced severe adverse events attributable to the MEK inhibitor treatment, with toxicities similar to what has been previously reported.

### DNA methylation profiling reveals a novel epigenetic group of NF1-associated pilocytic astrocytomas

**Fig. 5:**
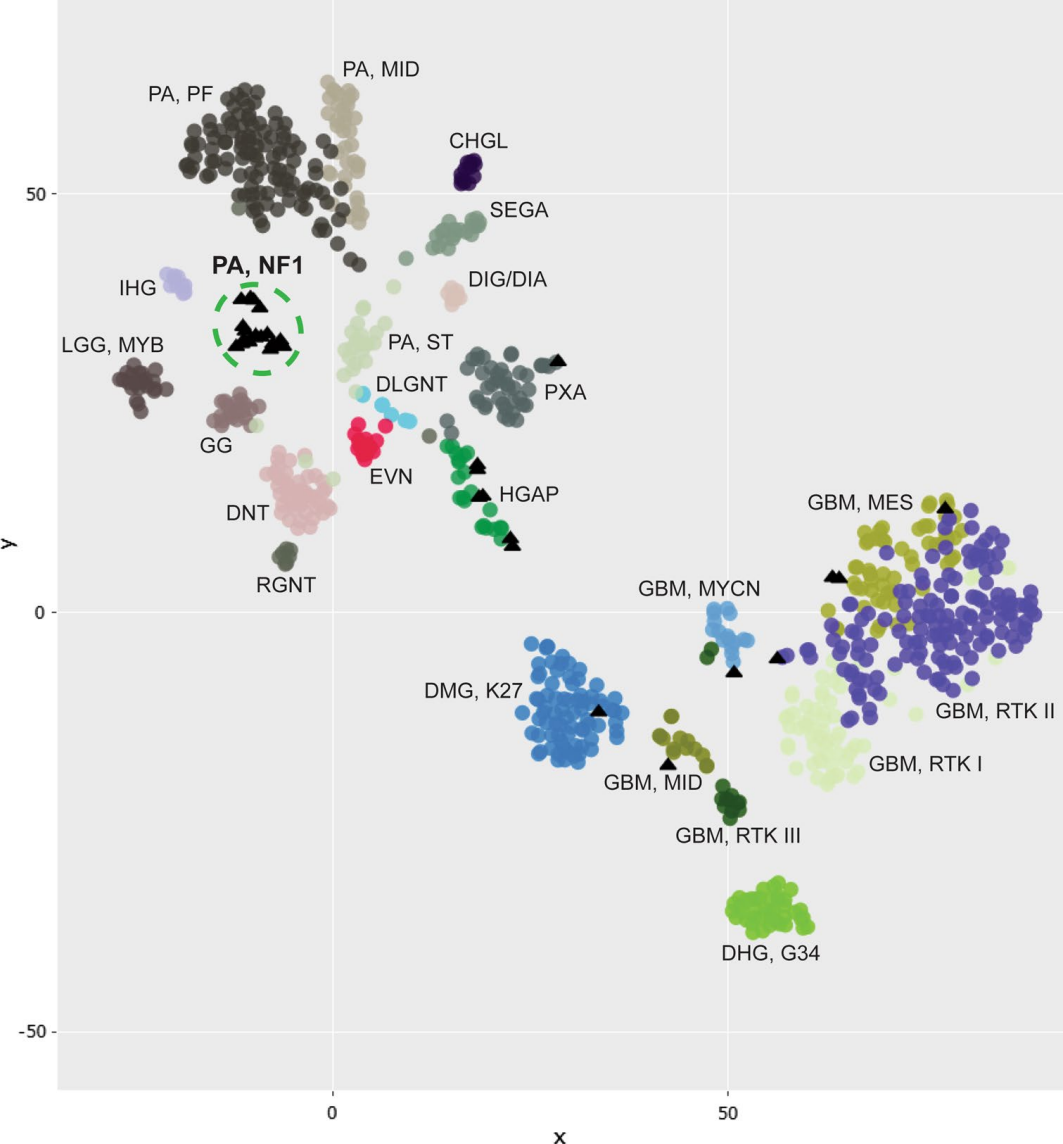
DNA methylation clustering analysis of 32 NF1-associated gliomas (black triangles), alongside a reference set of CNS tumor samples (circles). Shown is a two-dimensional representation of pairwise sample correlations using the 35,000 most variably methylated probes using t-distributed stochastic neighbor embedding (tSNE). Reference methylation classes depicted include: CHGL, chordoid glioma; DHG, G34, diffuse hemispheric glioma, H3 G34-mutant; DIG/DIA, desmoplastic infantile ganglioglioma/astrocytoma; DLGNT, diffuse leptomeningeal glioneuronal tumor; DMG, K27, diffuse midline glioma, H3 K27-altered; DNT, dysembryoplastic neuroepithelial tumor; EVN, extraventricular neurocytoma; GBM, MES, IDH-wildtype glioblastoma, mesenchymal subtype; GBM, MID, IDH-wildtype glioblastoma, midline subclass; GBM, MYCN, IDH-wildtype glioblastoma, MYCN subclass; GBM, RTK I IDH-wildtype glioblastoma, RTK I subclass; GBM, RTK II, IDH-wildtype glioblastoma, RTK II subclass; GBM, RTK III, IDH-wildtype glioblastoma, RTK III subclass; GG, ganglioglioma; HGAP, high-grade astrocytoma with piloid features; IHG, infant-type hemispheric glioma; LGG, MYB, low-grade glioma with *MYB* or *MYBL1* rearrangement; PA, MID, midline pilocytic astrocytoma; PA, PF, posterior fossa pilocytic astrocytoma; PA, ST, supratentorial/hemispheric pilocytic astrocytoma; PXA, pleomorphic xanthoastrocytoma; RGNT, rosette-forming glioneuronal tumor; SEGA, subependymal giant cell astrocytoma. See Supplementary Fig. 4 [Online Resource 2] for expanded tSNE plot and Supplementary Table 3 [Online Resource 1] for the sample manifest

Genome-wide DNA methylation profiling was performed on 32 NF1-associated gliomas from this patient cohort. None of the 18 tumors belonging to the molecular low-grade group classified with high calibrated scores (> 0.9) to any reference entities in version 11b4 of the online DKFZ random forest classifier, and only 5 of the 18 tumors classified as pilocytic astrocytoma (3 as infratentorial and 2 as midline subclasses) with high calibrated score in the newest version 12.5 of the classifier (Supplementary Table 10 [Online Resource 1]). However, all 18 of these NF1-associated gliomas belonging to the molecular low-grade group formed a distinct cluster separate from other recognized reference entities including sporadic pilocytic astrocytomas belonging to the supratentorial, midline, and posterior fossa subclasses based on t-distributed stochastic neighbor embedding (tSNE) and unsupervised hierarchical clustering analyses (Fig. 5, Supplementary Figs. 4–5 [Online Resource 2]). Co-embedding together with DNA methylation profiles from a previously reported cohort of 43 histologically low-grade gliomas arising in children with NF1 [7] demonstrated that the majority of those tumors clustered with the 18 molecular low-grade NF1-asociated gliomas from our study rather than the 3 reference subclasses of sporadic pilocytic astrocytoma (Supplementary Fig. 6 [Online Resource 2]).

### High-grade NF1-associated gliomas are epigenetically heterogeneous

The 14 NF1-associated gliomas belonging to the molecular high-grade group did not form a distinct epigenomic cluster but instead aligned with other sporadic reference entities, most frequently high-grade astrocytoma with piloid features (HGAP, 36%) and various subclasses of IDH-wildtype glioblastoma (43%) (Fig. 5, Supplementary Fig. 4 [Online Resource 2], Supplementary Table 10 [Online Resource 1]). The five tumors that clustered together with HGAP all had co-occurring *CDKN2A* homozygous deletion and *ATRX* truncating mutations. Among those that clustered with IDH-wildtype glioblastoma, four of the six tumors were with the mesenchymal subclass (all of which had co-occurring *CDKN2A* homozygous deletion but only one with co-occurring *ATRX* mutation), one was with the MYCN subclass (which had focal high level *MYCN* amplification), and the remaining one was with the midline subclass (which had truncating *SETD2* mutation). Only two of the six NF1-associated gliomas that clustered with IDH-wildtype glioblastoma had the combination of chromosome 7 gain and chromosome 10 loss, both of which aligned with the mesenchymal subclass. There were also individual gliomas that clustered with pleomorphic xanthoastrocytoma (which had co-occurring *BRAF* p.V600E mutation and *CDKN2A* homozygous deletion) and H3 K27-altered diffuse midline glioma (which had co-occurring *SUZ12* deletion, *TP53* mutation, and *ATRX* mutation) (Supplementary Fig. 7 [Online Resource 2]). Kaplan–Meier survival analysis revealed inferior outcomes for those patients with NF1-associated gliomas epigenetically aligning with HGAP or IDH-wildtype glioblastoma compared to those aligning with the novel NF1-associated pilocytic astrocytoma group (Supplementary Fig. 8 [Online Resource 2]).

### Longitudinal genomic analysis of clinically aggressive NF1-associated gliomas

**Fig. 6:**
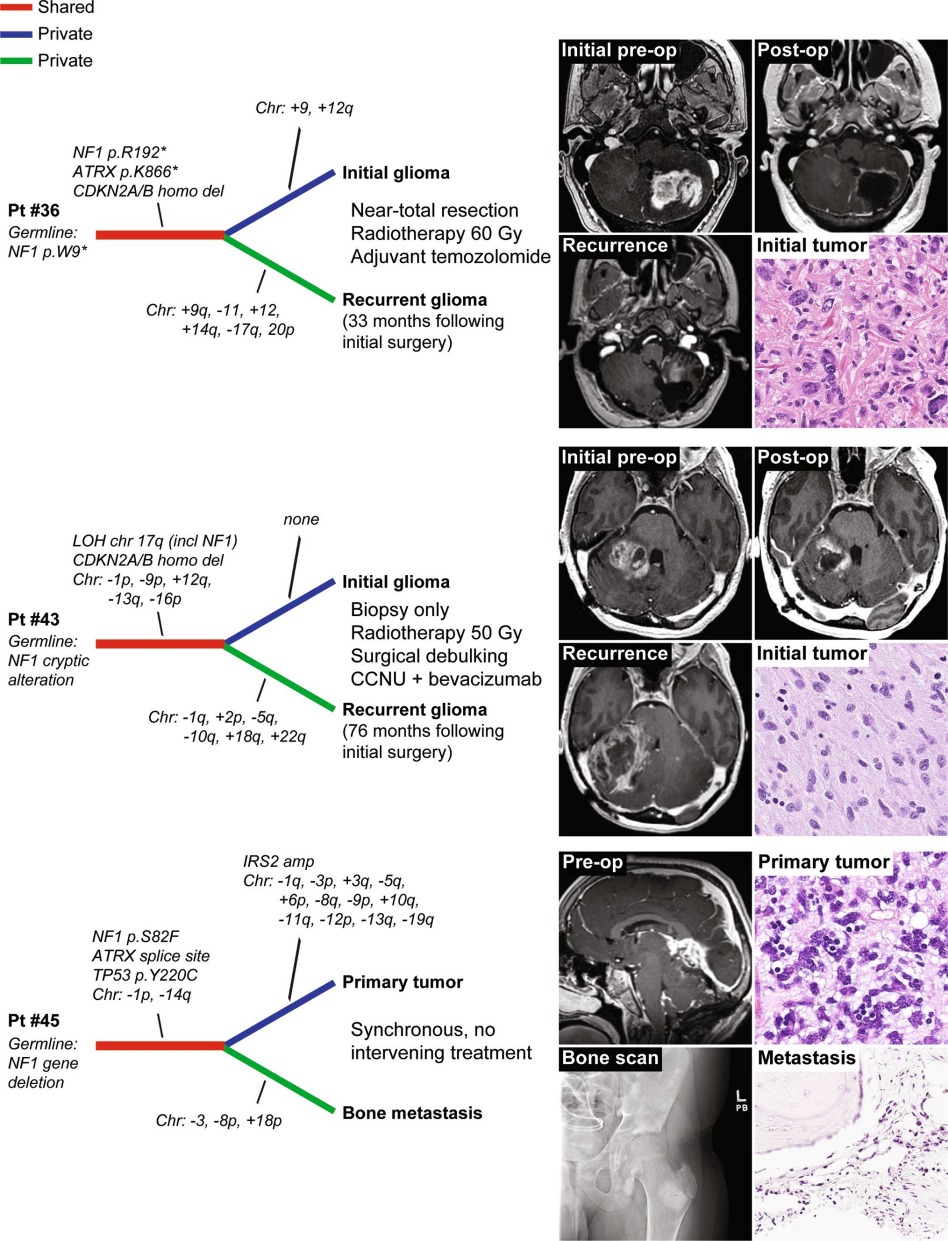
Longitudinal genomic analysis of gliomas arising in the setting of NF1. Shown are genetic evolution dendrograms generated from genomic analysis performed on two temporally or spatially distinct tumor specimens for each of three NF1 patients

Longitudinal genomic analysis of gliomas at time of diagnosis and recurrence following treatment has revealed important insight into the sequence of genetic alterations during gliomagenesis and the molecular mechanisms driving tumor progression and resistance to therapy. To investigate the genomic evolution of clinically aggressive NF1-associated gliomas, we performed genomic analysis on paired initial and recurrent glioma specimens for two patients, as well as a paired primary glioma and synchronous bone metastasis for one patient (Fig. 6, Supplementary Table 11 [Online Resource 1]). This demonstrated multiple chromosomal copy number gains and losses that were private to either the initial or recurrent/metastatic tumors, and an absence of newly acquired oncogenic drivers in the recurrent glioma specimens. Shared biallelic *NF1* inactivation was present in all three tumor pairs, as well as shared *CDKN2A* homozygous deletion and *ATRX* mutation in two tumor pairs each, thereby indicating these were initiating truncal events critical to gliomagenesis in the setting of NF1.

## Discussion

Neurofibromatosis type 1 (also commonly termed peripheral neurofibromatosis or von Recklinghausen’s disease) is a tumor predisposition syndrome that increases risk for a broad spectrum of neoplasms in multiple organ systems [15]. However, the predominant tumors are glial neoplasms of the central and peripheral nervous systems, including the namesake neurofibroma, with malignant gliomas and malignant peripheral nerve sheath tumors representing the two most common causes of cancer-related mortality for NF1 patients [21, 31]. Advances in understanding the molecular pathogenesis of the unique gliomas arising in this patient population are needed to improve prognostic classification and guide precision therapeutics.

**Fig. 7:**
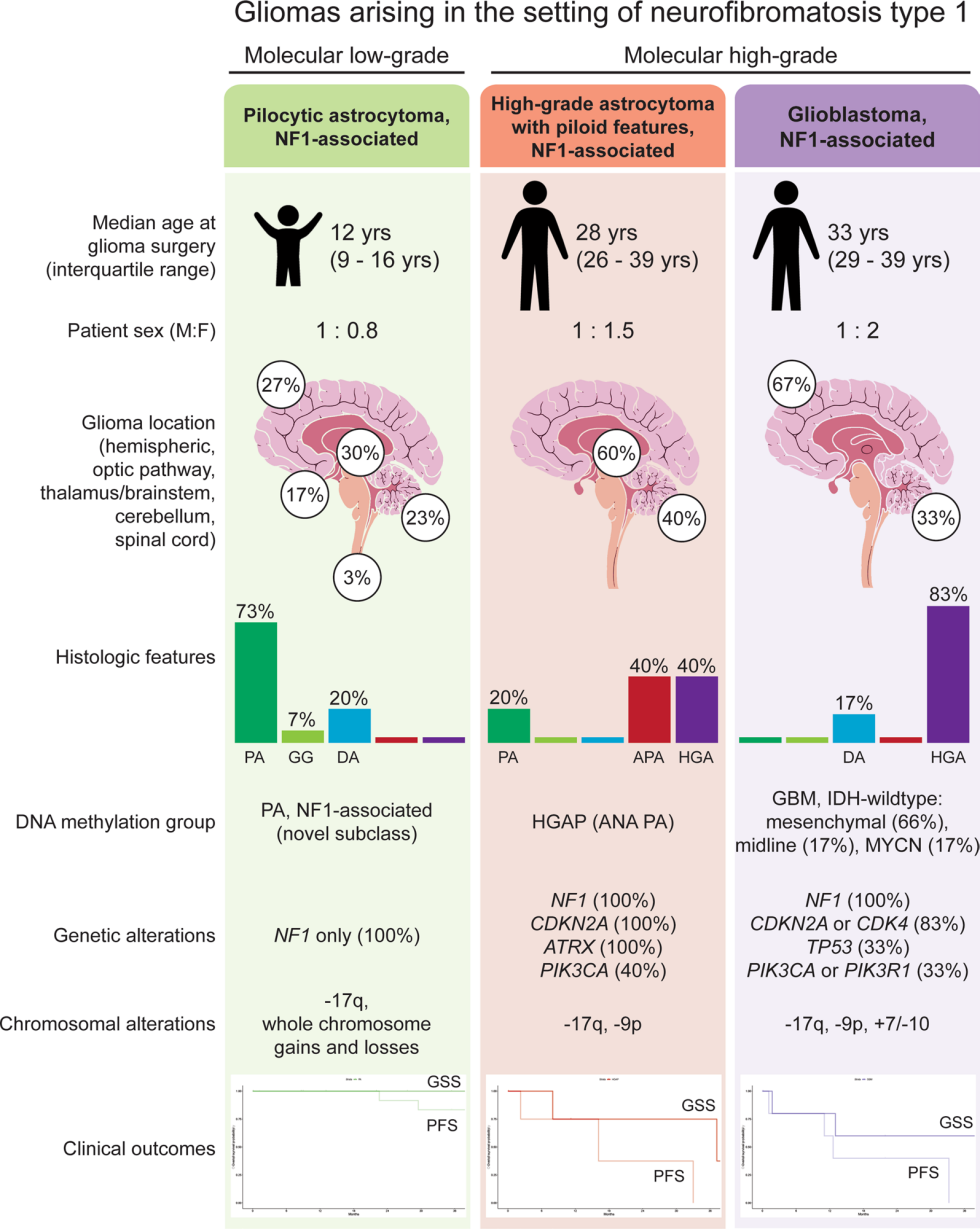
Graphical summary of the three main epigenetic classes of gliomas arising in the setting of neurofibromatosis type 1. PA, pilocytic astrocytoma. GG, ganglioglioma. DA, diffuse astrocytoma. APA, anaplastic pilocytic astrocytoma. HGA, high-grade astrocytoma. GSS, glioma-specific survival. PFS, progression-free survival

Through multiplatform molecular profiling, we identified two molecular groups of NF1-associated gliomas with divergent clinical outcomes (Fig. 7). The first occurs primarily during childhood, harbors biallelic *NF1* inactivation only, follows a more indolent clinical course, and has a unique epigenetic signature for which we propose the terminology “pilocytic astrocytoma, arising in the setting of NF1”. The other occurs primarily during adulthood, harbors additional oncogenic alterations including *CDKN2A* homozygous deletion and *ATRX* mutation, follows a more aggressive clinical course, and is epigenetically diverse, with most tumors aligning with either HGAP or various subclasses of IDH wild-type glioblastoma.

Low-grade gliomas arising in the setting of NF1 occur throughout the neuroaxis with a large proportion arising in the optic pathway, cerebellum, and thalamus. Historically, they have been variably diagnosed as pilocytic astrocytoma, ganglioglioma, or diffuse astrocytoma [24]. However, these syndromic tumors have a different genetic driver within the MAP kinase pathway compared to most sporadic pilocytic astrocytomas and gangliogliomas which typically harbor *BRAF* fusion or mutation. Intriguingly, we have identified that our cohort of NF1-associated low-grade gliomas resolve into a single epigenetic cluster, despite occurring at different anatomic sites and having variable histologic features ranging from conventional pilocytic astrocytoma to those with an infiltrative growth pattern resembling diffuse astrocytoma or with a ganglion cell component resembling ganglioglioma. These epigenetic results are similar to a recent study that found most NF1-associated low-grade gliomas did not cluster together with the three reference classes of sporadic pilocytic astrocytomas (supratentorial, midline, and posterior fossa) [7]. Our re-analysis of DNA methylation profiles from this prior NF1-associated low-grade glioma cohort found that the majority aligned with our novel methylation subclass of NF1-associated pilocytic astrocytomas. As such, we believe NF1-associated low-grade gliomas should be regarded as a distinct biologic entity and henceforth classified as “pilocytic astrocytoma, arising in the setting of NF1”. Prospectively, DNA methylation testing revealing that a patient’s glioma epigenetically aligns with this group should prompt consideration of underlying NF1.

While biallelic inactivation of the *NF1* tumor suppressor gene is sufficient to drive gliomagenesis of low-grade gliomas in both humans and mice [2, 27, 34], additional genetic aberrations disrupting the cell cycle and enabling telomere maintenance appear to be fundamental to the development of malignant high-grade gliomas in patients with NF1. Of the 17 gliomas in our molecular high-grade group, 82% had either *CDKN2A* inactivation (*n* = 13) or *CDK4* amplification (*n* = 1). Similar to recent studies demonstrating alternative lengthening of telomeres (ALT) driven by *ATRX* inactivation in NF1-associated malignancies [4, 25], 53% of the gliomas in our high-grade molecular group (9/17) had inactivating *ATRX* mutations, with uniform absence of *TERT* promoter mutation or other *TERT* alterations. Less common genetic drivers of NF1-associated high-grade gliomas included *TP53* mutation (3/17, 18%), *PIK3CA* or *PIK3R1* mutation (4/17, 24%), *PTEN* mutation (1/17, 6%), *PDGFRA* amplification (1/17, 6%), *MYCN* amplification (1/17, 6%), *PPM1D* mutation (1/17, 6%), and *SETD2* mutation (1/17, 6%). Together, these cooperating genetic alterations may represent additional therapeutic targets beyond MEK inhibition for NF1-associated high-grade gliomas, specifically using small molecule inhibitors of CDK4/6 (*e.g.,* abemaciclib) and either PARP (*e.g.,* olaparib) or ATR (*e.g.,* berzosertib) given the known synthetic lethality in tumors that utilize alternative lengthening of telomeres [8, 10, 33].

In contrast to the molecular low-grade group that formed a single distinct epigenetic cluster, we found that NF1-associated high-grade gliomas are epigenetically diverse, with most cases aligning with either HGAP or various subclasses of IDH-wildtype glioblastoma. High-grade astrocytoma with piloid features (HGAP) is a recently described subtype of high-grade glioma that is characterized by a unique epigenetic signature, MAP kinase pathway activation (most often involving *NF1*, *FGFR1*, or *BRAF*) along with the combination of *CDKN2A* deletion and *ATRX* mutation, and an unfavorable clinical course intermediate between IDH-mutant gliomas and IDH-wildtype glioblastoma [23]. Our study has revealed that a subset of HGAP arise in the setting of NF1, and that these NF1-associated HGAP have overlapping molecular features with their sporadic counterparts including the trio of *NF1* inactivation, *CDKN2A* homozygous deletion, and *ATRX* mutation. Our cohort of NF1-associated HGAP exhibited variable morphology, including some with Rosenthal fibers and fine fibrillar processes, while others lacked these piloid features. In contrast, while 5/6 NF1-associated gliomas epigenetically aligning with IDH-wildtype glioblastoma harbored either *CDKN2A* homozygous deletion or *CDK4* amplification similar to NF1-associated HGAP, only one of these six tumors had *ATRX* mutation, and none exhibited piloid features. Notably, 4/6 of the NF1-associated glioblastomas from our study epigenetically aligned with the mesenchymal subclass of glioblastoma, which is the epigenetic subclass that encompasses most sporadic glioblastomas with somatic *NF1* inactivation [28, 32]. Only 2/6 of the NF1-associated glioblastomas from our study had the combination of trisomy 7 and monosomy 10, and none harbored *TERT* promoter mutation or *EGFR* amplification. The NF1-associated gliomas epigenetically clustering with either HGAP or IDH-wildtype glioblastoma had worse outcomes compared to the novel NF1-associated pilocytic astrocytoma methylation class, but appeared to have better prognosis than sporadic IDH-wildtype glioblastoma.

Consistent with an autosomal dominant tumor predisposition syndrome, we found that NF1-associated gliomas occurred in patients with a heterozygous germline (or mosaic) mutation/deletion involving one of two *NF1* alleles with tumors that developed following somatic inactivation of the remaining wildtype allele through either loss of heterozygosity or a second tumor-acquired mutation. Interestingly, 11 of the 47 gliomas in our study cohort demonstrated two or more somatic events contributing to inactivation of the remaining wildtype *NF1* allele. This suggests that glioma initiation may occur prior to biallelic *NF1* inactivation in some patients, with the somatic events targeting the remaining wildtype *NF1* allele occurring within different tumor subclones later during gliomagenesis. This phenomenon of multiple different subclonal somatic events contributing to the biallelic inactivation of a tumor suppressor gene has not been previously described in other tumor predisposition syndromes to the best of our knowledge, and the clinical implications of this intriguing observation about the origins of NF1-associated gliomas remains to be defined.

Relatedly, clonal evolution of NF1-associated gliomas and the mechanisms of tumor progression and treatment resistance remains poorly understood. No patients in our study with tumors belonging to the molecular high-grade group had a prior pathologically confirmed lower-grade precursor in the same anatomic location. Whether the high-grade gliomas arising in the setting of NF1 occur de novo or from transformation of a pre-existing low-grade glioma following acquisition of additional oncogenic drivers (*e.g., CDKN2A* deletion, *ATRX* mutation) remains uncertain. However, given that these NF1-associated high-grade gliomas occur almost exclusively in adults and not during childhood, we speculate that a subset of these tumors evolve from transformation of a lower-grade precursor over time, perhaps specifically those with piloid features and a DNA methylation signature aligning with HGAP given the overlapping histologic features with pilocytic astrocytoma? In our longitudinal analysis of three paired initial and recurrent or metastatic high-grade gliomas, the key genetic drivers (*e.g., NF1*, *CDKN2A*, *ATRX*) were shared truncal events while only chromosomal copy number aberrations were private to initial or recurrent/metastatic tumors. In both patients who received radiotherapy and alkylating chemotherapy, there was an increased burden of chromosomal gains/losses in the recurrent gliomas but no increased single nucleotide variant load or additional oncogenic drivers beyond cytogenetic aberrations. Whether these additional chromosomal gains and losses (which were non-overlapping between the two patients) represent therapy-related changes or a selectively acquired resistance mechanism requires further study.

Since gliomas are one of the leading causes of mortality in patients with NF1, continued development of efficacious treatment strategies is needed. As these and other NF1-associated tumors uniformly harbor biallelic *NF1* inactivation that results in activation of Ras-Raf-MEK-ERK signaling, inhibition of this MAP kinase pathway has been explored as a therapeutic strategy in preclinical models [17]. Selumetinib is a small molecule MEK inhibitor now approved by the United States Food and Drug Administration and European Medicines Agency for the treatment of symptomatic, surgically unresectable plexiform neurofibromas following demonstration of significant efficacy with durable tumor shrinkage in clinical trials [5, 11], and clinical trials investigating the efficacy of selumetinib in treating NF1-associated low-grade gliomas are ongoing [6]. Targeting the PI3-kinase-Akt-mTOR signaling pathway with the mTOR small molecule inhibitor everolimus has also shown promising efficacy in a phase II clinical trial for children with recurrent/progressive NF1-associated low-grade gliomas [30]. In our cohort, all five NF1 patients with molecular low-grade gliomas that were treated with small molecule MEK inhibitors (either selumetinib or trametinib) experienced stable disease or tumor regression and did not require additional therapies, whereas patients with molecular high-grade gliomas did not respond to single agent MEK inhibition. While single agent MEK or mTOR inhibition remains a promising strategy for NF1-associated gliomas belonging to the molecular low-grade group with biallelic *NF1* inactivation only, those NF1-associated gliomas with additional oncogenic alterations belonging to the molecular high-grade group likely require combinatorial treatment approaches to be evaluated in future clinical trials.

Given our findings, we advocate for prospective molecular testing on gliomas arising in the setting of NF1 to stratify them into prognostically and therapeutically relevant low-grade versus high-grade molecular groups, and DNA methylation profiling to accurately classify high-grade tumors into HGAP, IDH-wildtype glioblastoma, or other subtypes. We also recommend adding “arising in the setting of neurofibromatosis type 1” to the integrated pathologic diagnosis for this family of syndromic gliomas to distinguish them from their sporadic counterparts, which can be modified with the descriptors of “clinically diagnosed” versus “genetically confirmed” dependent on the clinical scenario. Additional studies and clinical trials are needed to further define the underlying biologic nature of NF1-associated gliomas and determine optimal treatment strategies for affected patients.

## Supplementary Information

Below is the link to the electronic supplementary material.

Supplementary file1 (XLSX 114 KB)

Supplementary file2 (PDF 13647 KB)

## Data Availability

Digitally scanned image files of representative H&E and immunostained sections are available at the following link: https://figshare.com/projects/Gliomas_arising_in_the_setting_of_neurofibromatosis_type_1/141068. DNA methylation array data files are available from the Gene Expression Omnibus (GEO) repository under accession number GSE198656 (https://www.ncbi.nlm.nih.gov/geo/). Annotated DNA sequencing data are available in the electronic supplementary material. Raw sequencing data files are available upon request.
