# Supplementary material for: Multiplatform molecular analyses refine classification of gliomas arising in patients with neurofibromatosis type 1
Source: Acta Neuropathol. 2022 Aug 9;144(4):747–65. doi: 10.1007/s00401-022-02478-5 (PMC9468105; doi:10.1007/s00401-022-02478-5)
Supplement: Supplementary file 2 — Supplementary file2 (PDF 13647 KB) [file 401_2022_2478_MOESM2_ESM.pdf]

**Glioma arising in the setting of NF1, low-grade molecular group**

Pt #5; 1 y/o M; optic pathway tumor; *NF1* bialleleic inactivation only

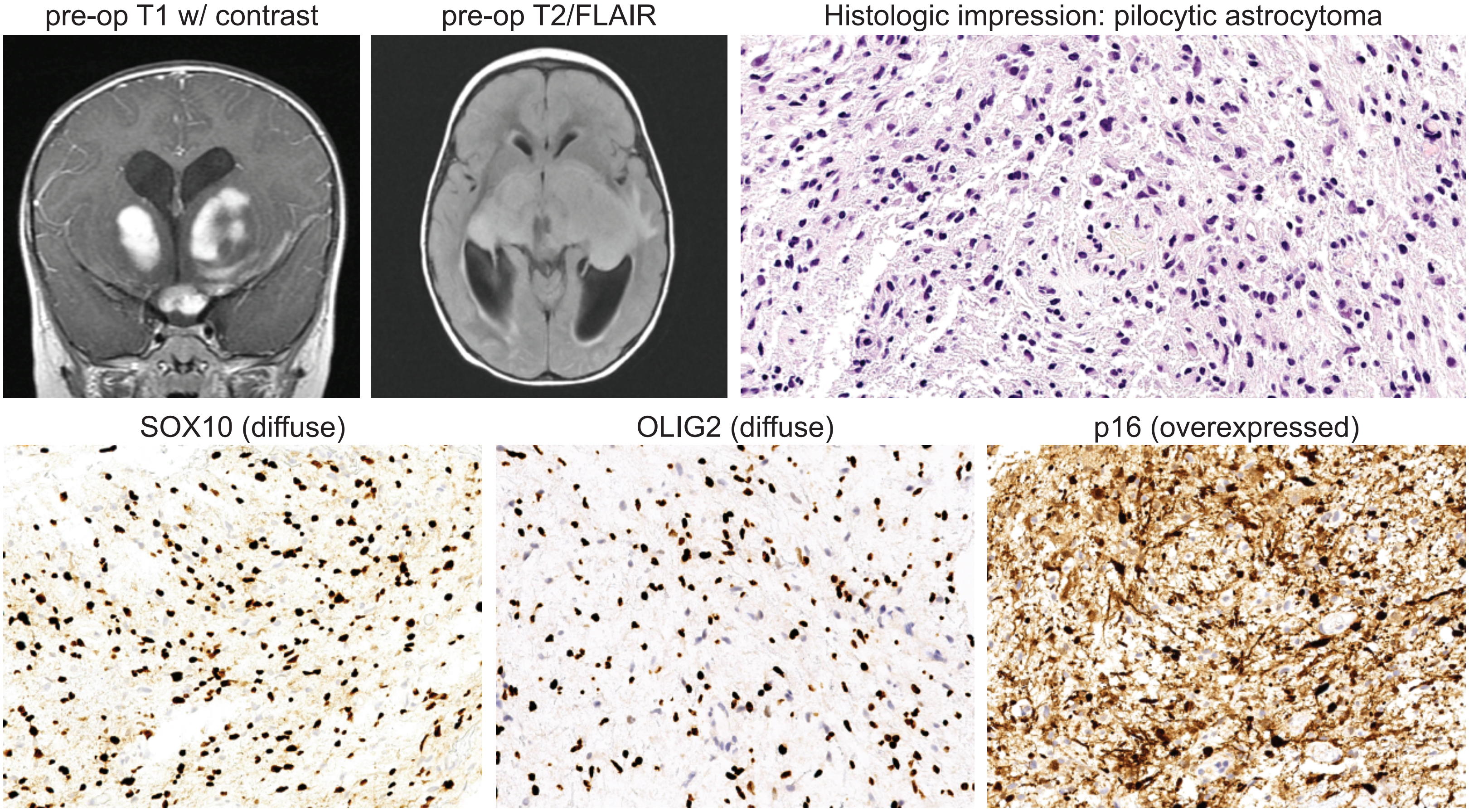


**Glioma arising in the setting of NF1, low-grade molecular group**

Pt #29; 9 y/o F; cerebellar tumor; *NF1* bialleleic inactivation only

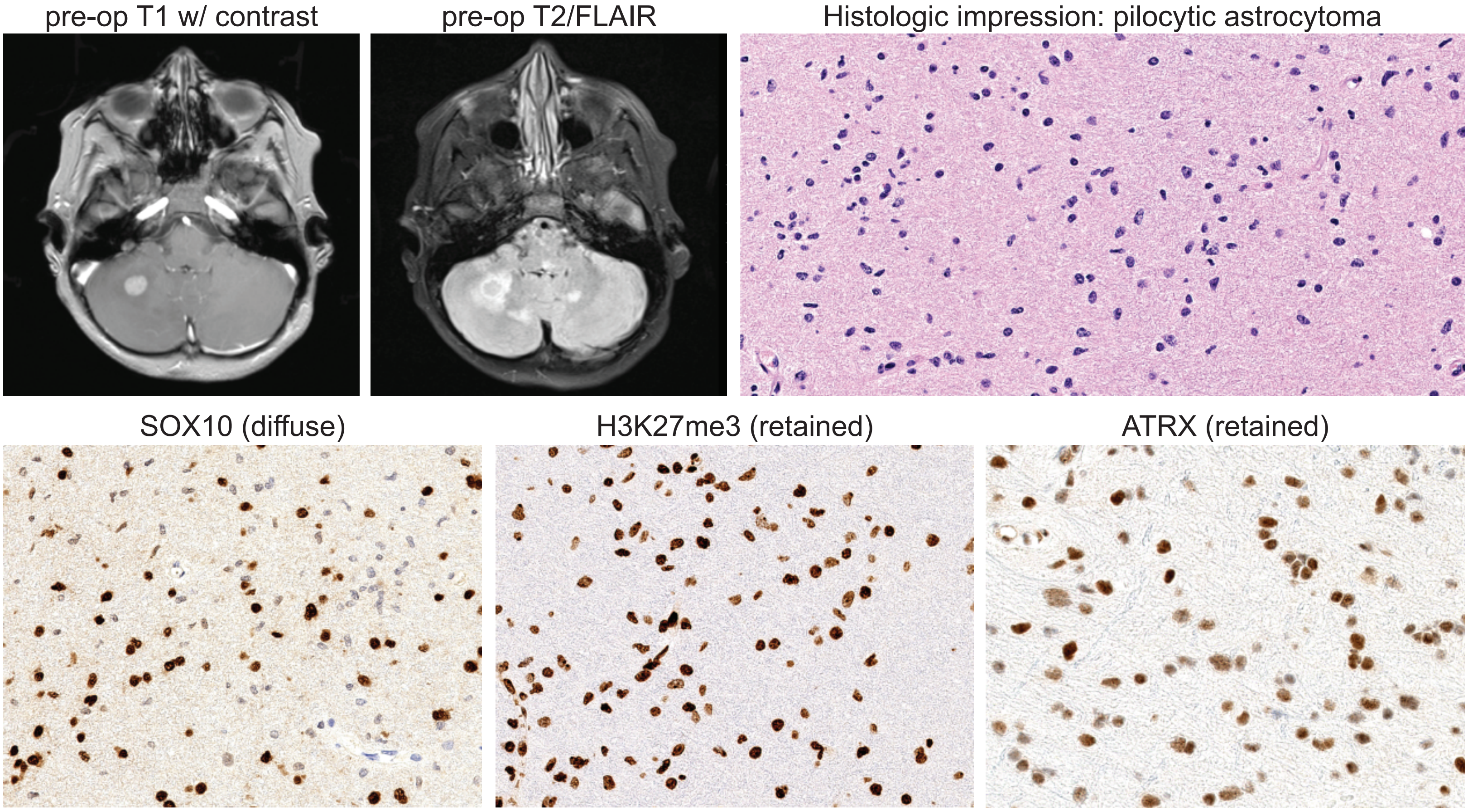


**Supplementary Figure 1.** Imaging and histologic features of gliomas arising in patients with NF1. Pilocytic astrocytoma, NF1-associated, occurring in the optic pathway (patient #5, top). Pilocytic astrocytoma, NF1-associated, occurring in the cerebellum (patient #29, bottom).

**Glioma arising in the setting of NF1, low-grade molecular group**

Pt #9; 5 y/o F; cerebral tumor; *NF1* bialleleic inactivation only; DNA me3 = PA, NF1-associated

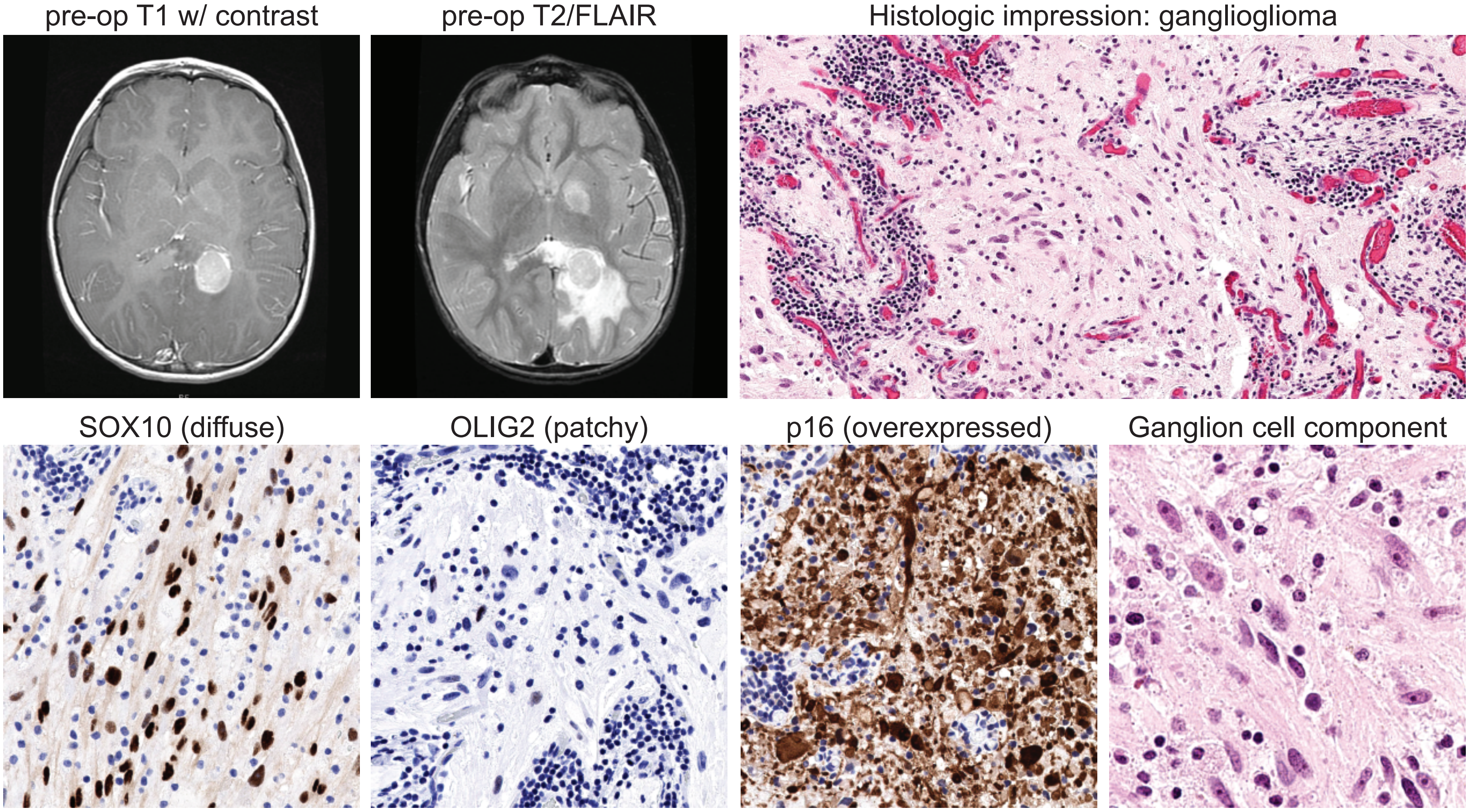


**Glioma arising in the setting of NF1, low-grade molecular group**

Pt #19; 15 y/o M; brainstem tumor; *NF1* biallelic inactivation only; DNA me3 = PA, NF1-associated

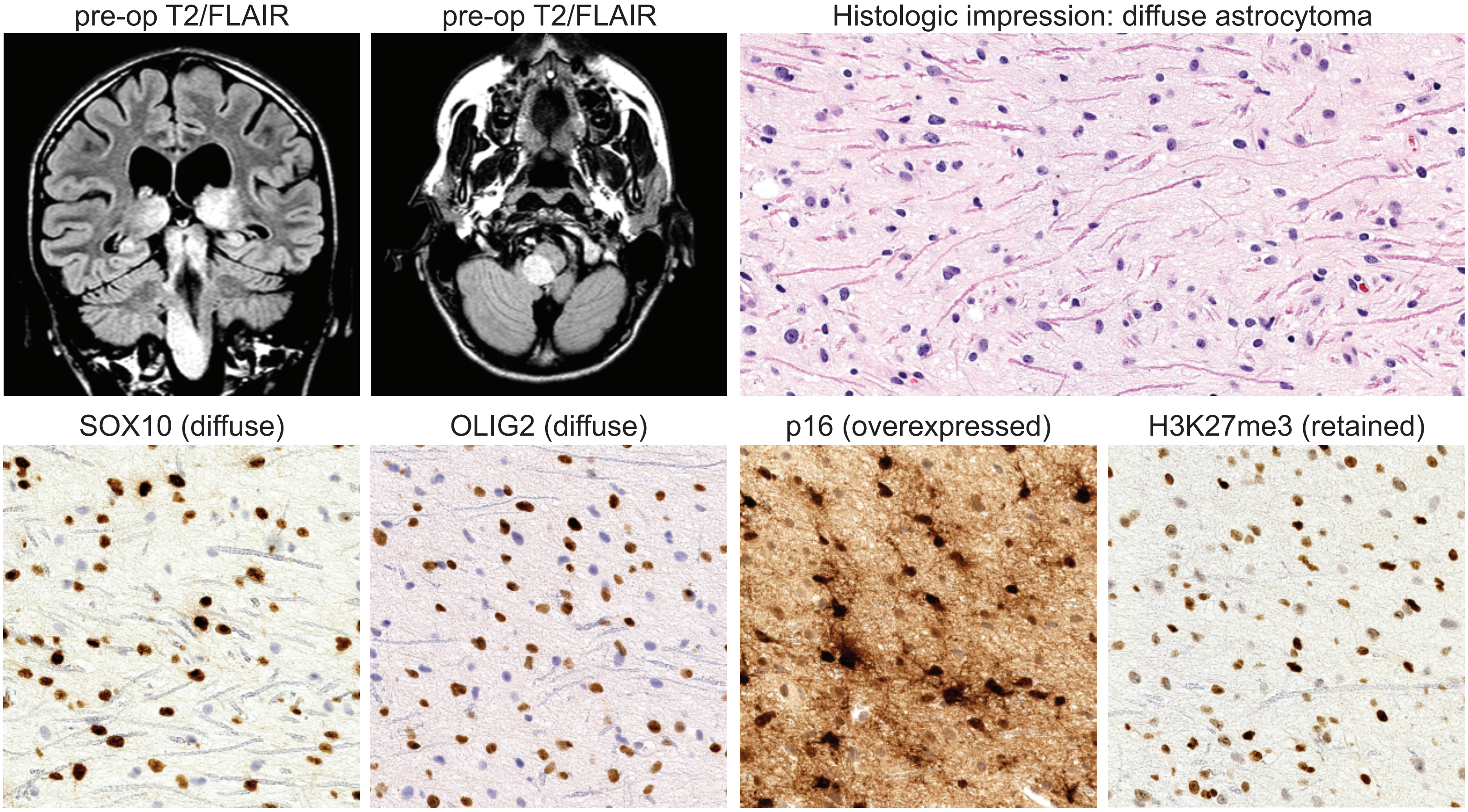


**Supplementary Figure 2.** NF1-associated gliomas belonging to the new epigenetic class of “Pilocytic astrocytoma, NF1-associated” include occasional tumors with histologic features resembling ganglioglioma and diffuse astrocytoma.

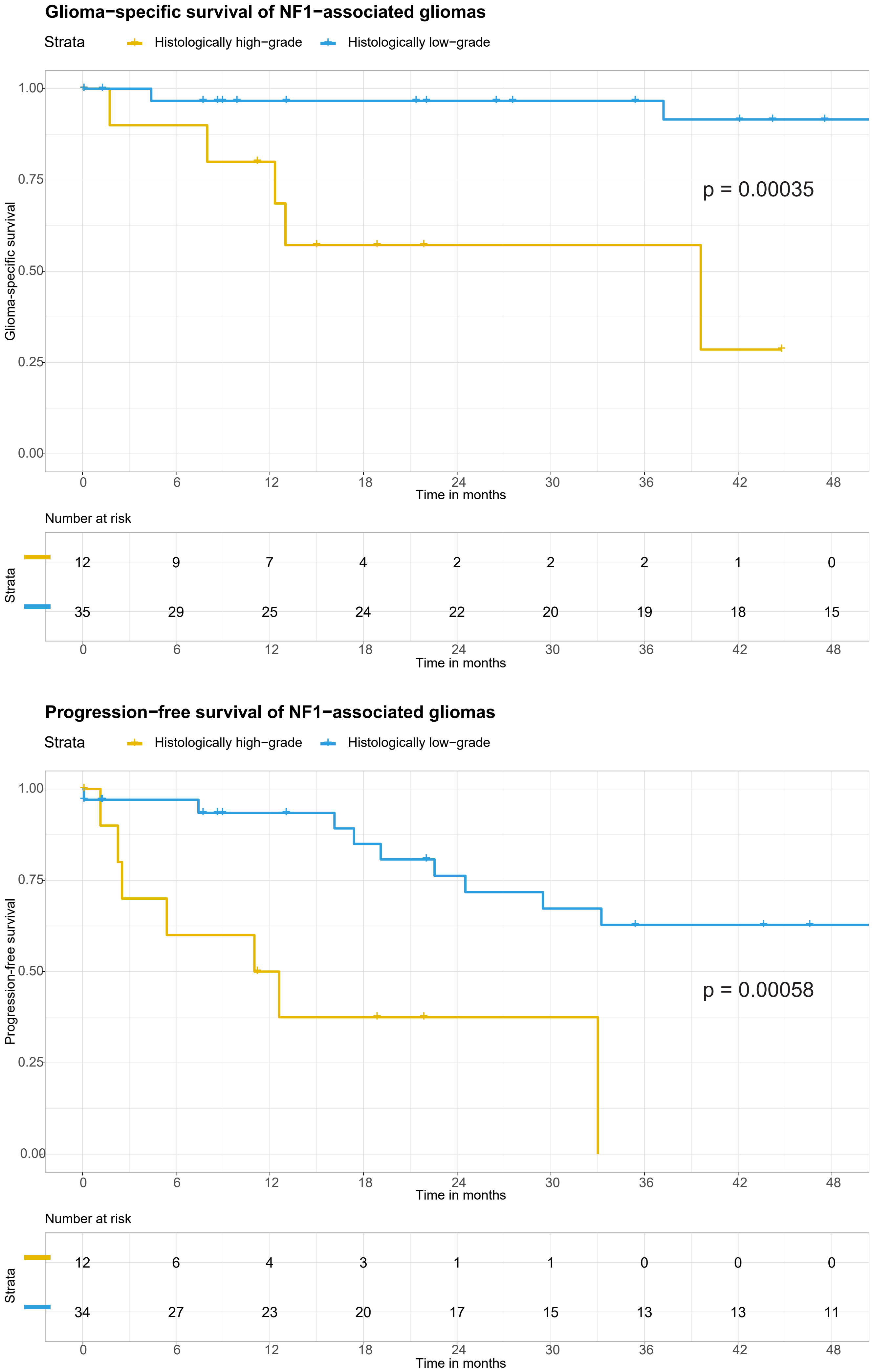


**Supplementary Figure 3.** Kaplan-Meier curves comparing glioma-specific survival (above) and progression-free survival (below) for the 47 NF1 patients stratified by tumor histologic features.

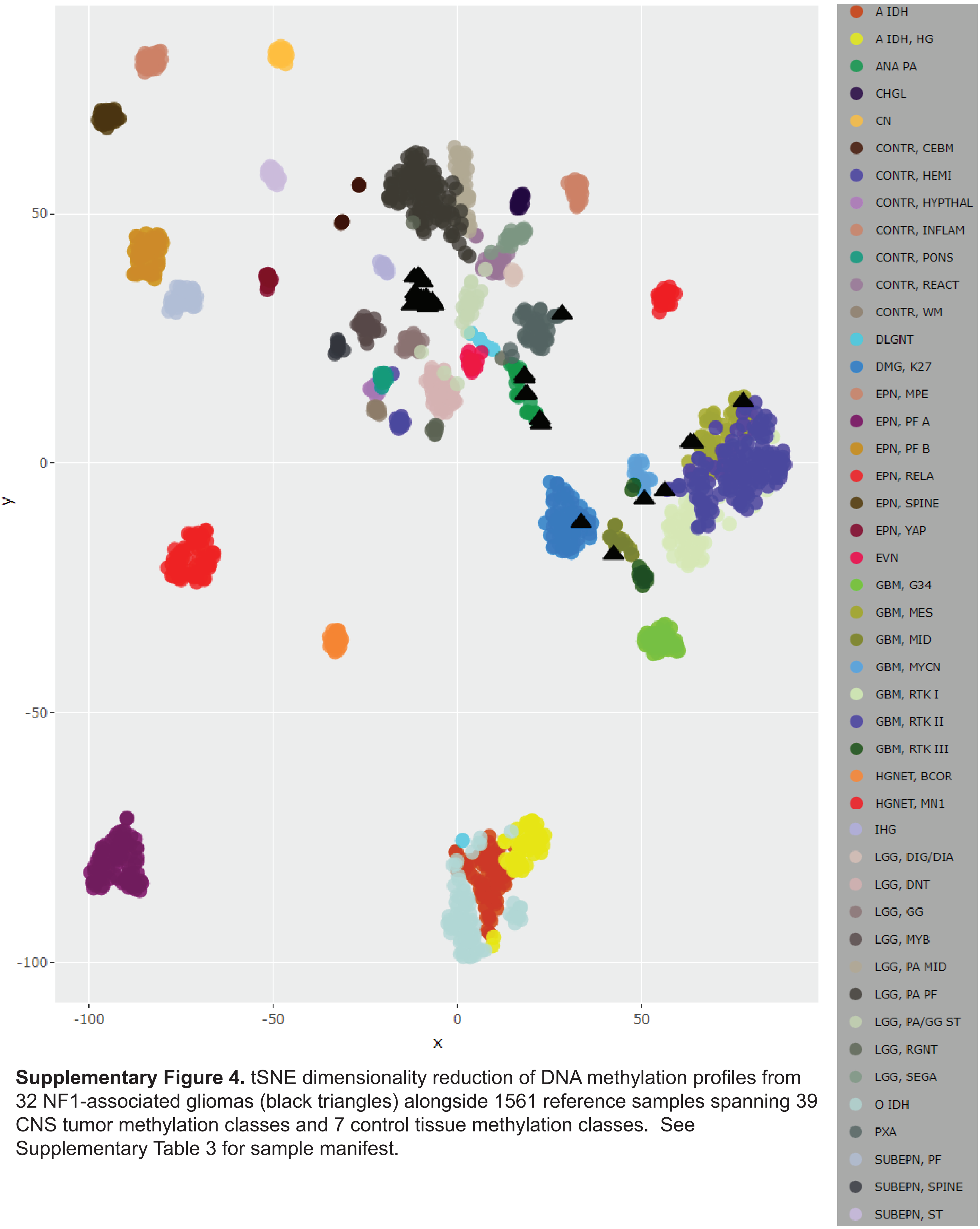


**Supplementary Figure 4.** tSNE dimensionality reduction of DNA methylation profiles from 32 NF1-associated gliomas (black triangles) alongside 1561 reference samples spanning 39 CNS tumor methylation classes and 7 control tissue methylation classes. See Supplementary Table 3 for sample manifest.

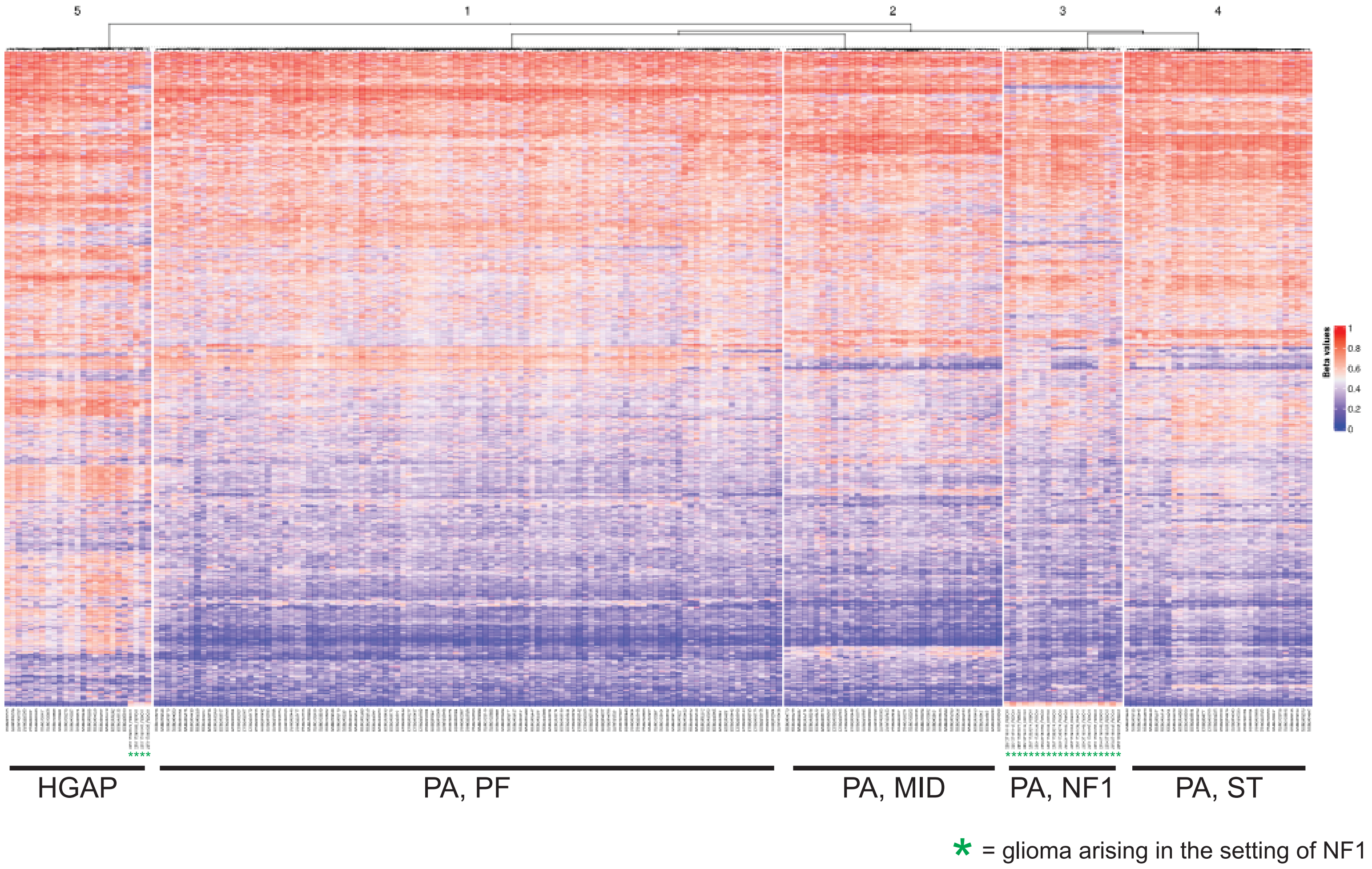


**Supplementary Figure 5.** Unsupervised hierarchical clustering of DNA methylation profiles from 24 NF1-associated gliomas alongside reference cohorts of high-grade astrocytoma with piloid features (HGAP), posterior fossa subclass of pilocytic astrocytoma (PA, PF), midline subclass of pilocytic astrocytoma (PA, MID), and supratentorial subclass of pilocytic astrocytoma (PA, ST). See Supplementary Table 4 for sample manifest.

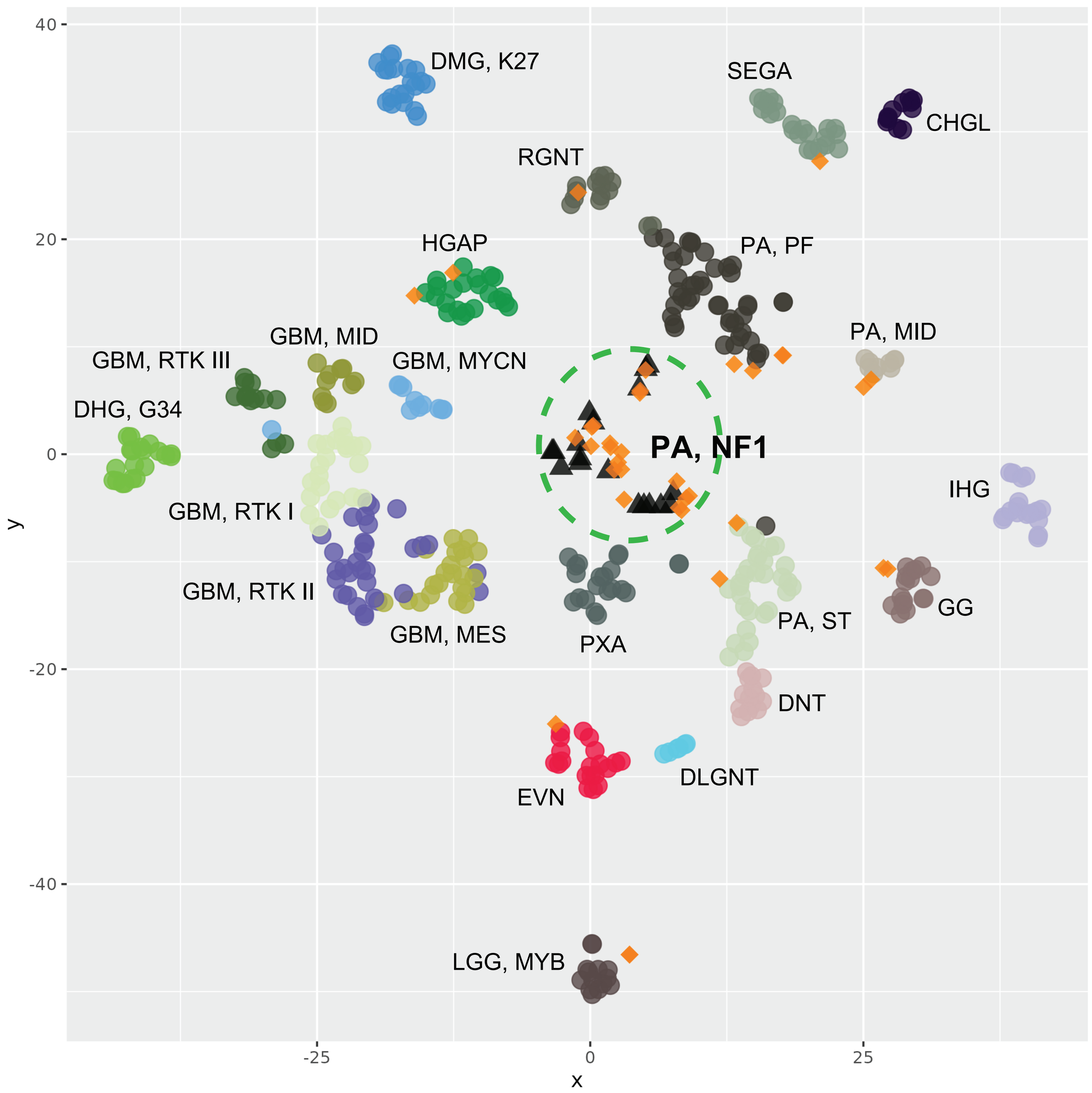


**Supplementary Figure 6.** DNA methylation clustering analysis of 18 NF1-associated pilocytic astrocytomas from this study (black triangles) and 43 histologically low-grade gliomas arising in children with NF1 (orange diamonds) from the Synodos NF1 LGG study (Fisher et al. *Acta Neuropath* 2021, 141(4):605-617, Synapse ID: syn5698493), alongside a reference set of CNS tumor samples (circles). Shown is a two-dimensional tSNE plot generated from the 25,000 most variably methylated probes with the following parameters: perplexity = 10, theta = 0.5, max_iter = 1000. Reference methylation classes depicted include: CHGL, chordoid glioma; DHG, G34, diffuse hemispheric glioma, H3 G34-mutant; DLGNT, diffuse leptomeningeal glioneuronal tumor; DMG, K27, diffuse midline glioma, H3 K27-altered; DNT, dysembryoplastic neuroepithelial tumor; EVN, extraventricular neurocytoma; GBM, MES, glioblastoma, mesenchymal subtype; GBM, MID, glioblastoma, midline subclass; GBM, MYCN, glioblastoma, MYCN subclass; GBM, RTK I glioblastoma, RTK I subclass; GBM, RTK II, glioblastoma, RTK II subclass; GBM, RTK III, glioblastoma, RTK III subclass; GG, ganglioglioma; HGAP, high-grade astrocytoma with piloid features; IHG, infant-type hemispheric glioma; LGG, MYB, low-grade glioma with *MYB*/*MYBL1* rearrangement; PA, MID, midline pilocytic astrocytoma; PA, PF, posterior fossa pilocytic astrocytoma; PA, ST, supratentorial/hemispheric pilocytic astrocytoma; PXA, pleomorphic xanthoastrocytoma; RGNT, rosette-forming glioneuronal tumor; SEGA, subependymal giant cell astrocytoma.

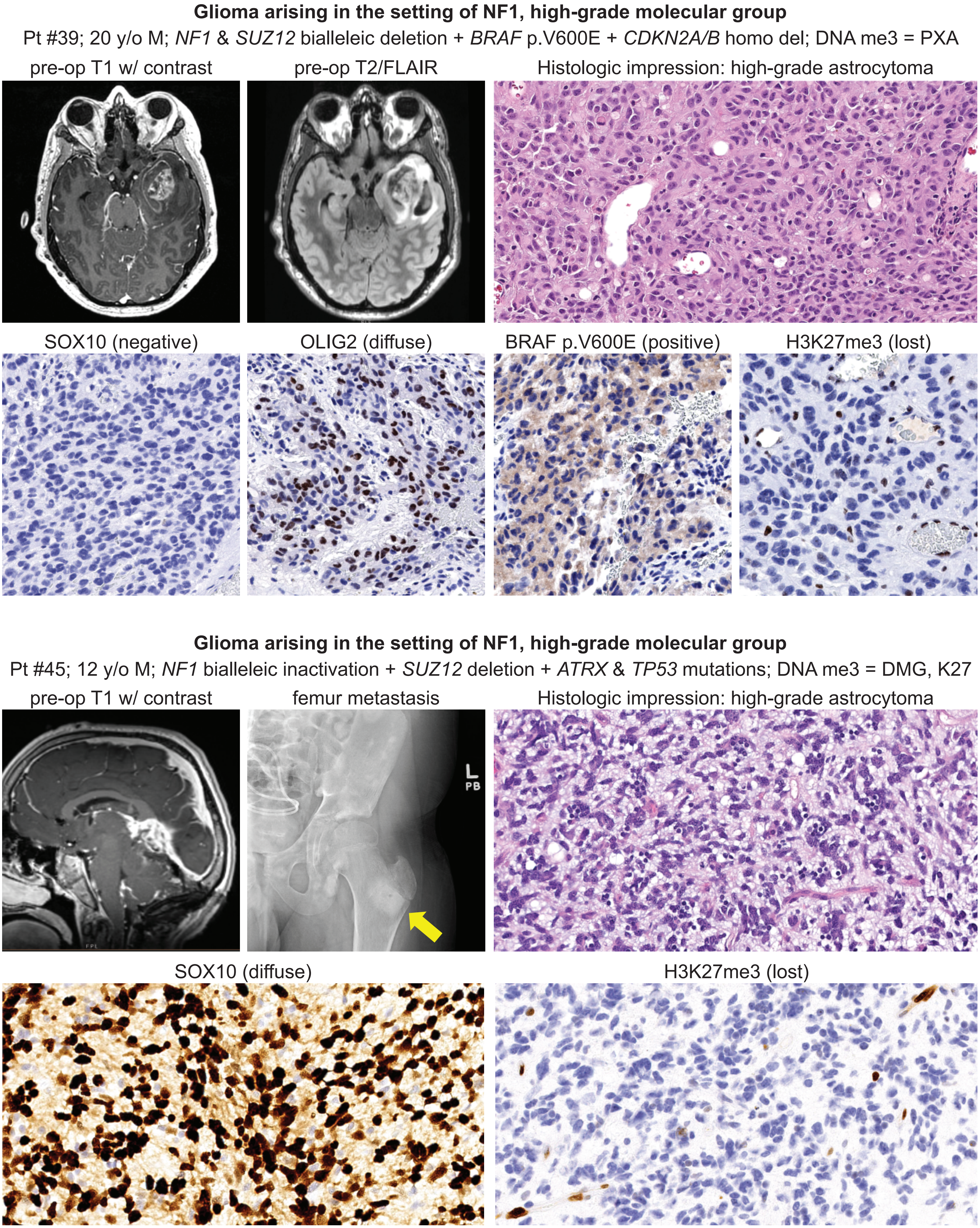


**Supplementary Figure 7.** NF1-associated gliomas belonging to the high-grade molecular group with additional oncogenic alterations beyond *NF1* inactivation include rare tumors epigenetically aligning with either pleomorphic xanthoastrocytoma (PXA) or H3 K27-altered diffuse midline glioma (DMG, K27).

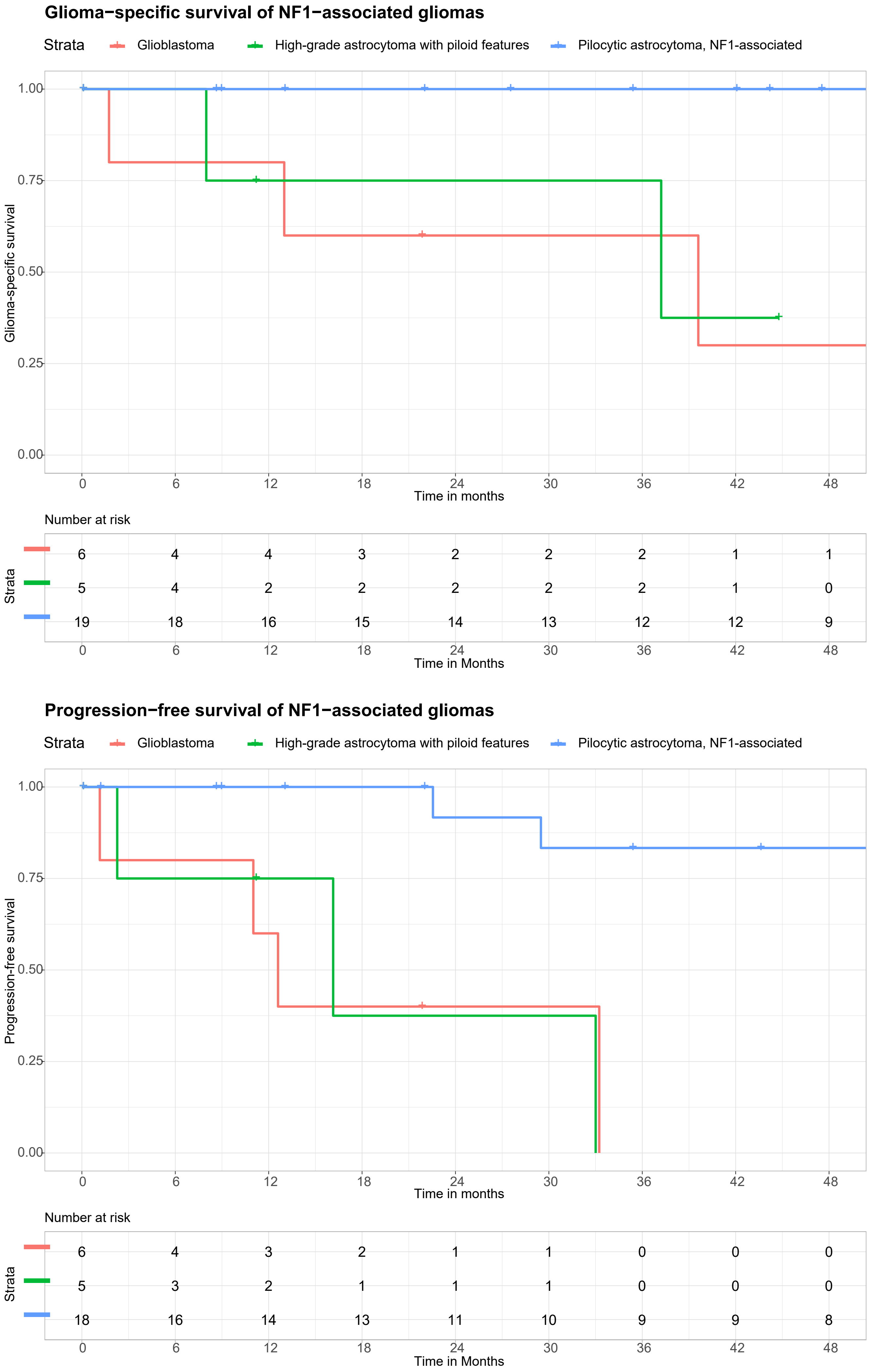


**Supplementary Figure 8.** Kaplan-Meier curves comparing glioma-specific survival (above) and progression-free survival (below) for 30 NF1 patients stratified by epigenomic tumor classification.
